# Supplementary material for: Generalised anxiety disorder and hospital admissions: findings from a large, population cohort study
Source: BMJ Open. 2018 Oct 27;8(10):e018539. doi: 10.1136/bmjopen-2017-018539 (PMC6224748; doi:10.1136/bmjopen-2017-018539)
Supplement: Supplementary file 1 [file bmjopen-2017-018539supp001.pdf]

Appendix I: Table 1 Percentage and number of people with missing past-year GAD reported in 1996-2000 according to sociodemographic factors, health status, and behaviour risk factors for the EPIC-Norfolk cohort

	Total number with characteristic	Percentage and no. with missing past-year GAD
**Characteristic**		
**Socio-demographics**		
**Age (years)**		
<50	2385	1.1 (26)
50-60	6279	1.1 (70)
60-70	5787	0.9 (54)
70+	3685	1.3 (47)
**Sex**		
Women	10055	1.2 (118)
Men	8081	1.0 (79)
**Education**[‡]		
Low	6178	1.2 (72)
High	11958	1.1 (125)
**Marital status**		
Single	695	1.3 (9)
Married	14687	1.0 (149)
Other[*]	2754	1.4 (39)
**Social class**		
Manual	6918	1.2 (82)
Non-manual	11218	1.0 (115)
**Employment**		
Yes	7775	0.8 (63)[b]
No	10361	1.3 (134)
**Health status**		
**Physical conditions**[+]		
Yes	9285	1.3 (119)[b]
No	8851	0.9 (78)
**Disability level**		
High[¶]	9030	1.4 (130)[a]
Low	9106	0.7 (67)
**Psychiatric conditions**		
**Past-year MDD**		
Yes	983	5.0 (49)[a]
No	17153	0.9 (148)
**Behaviour risk factors**		
**Physical activity**		
Active[¥]	12963	1.1 (141)
Inactive	5173	1.1 (56)
**Smoking status**		
Current smoker	1922	1.5 (29)
Former smoker	7543	1.0 (73)

Never smoker	8671	1.1 (95)
**Alcohol intake**		
High[α]	9327	0.9 (86)^{b}
Low	8809	1.3 (111)

‡ High education: O-level, A-level, degree; low education: refers to no education

* Other: divorced, separated, widowed

+ Physical conditions: respiratory disease (asthma and bronchitis), allergies and hay fever, stroke, heart attack, cancer, diabetes, thyroid conditions, arthritis

¶ Below the median PCS value of 50.6

¥ Moderately inactive, moderately active, active

α 3+ units of alc./week (1 pint beer=2 units, 1 glass wine=1 unit, 1 glass sherry=1 unit, 1 glass spirits=1 unit)

^{a} $P < 0.001$

^{b} $P < 0.05$
