# Supplementary material for: Generalised anxiety disorder and hospital admissions: findings from a large, population cohort study
Source: BMJ Open. 2018 Oct 27;8(10):e018539. doi: 10.1136/bmjopen-2017-018539 (PMC6224748; doi:10.1136/bmjopen-2017-018539)
Supplement: Author's manuscript [file bmjopen-2017-018539.draft_revisions.pdf]

BMJ Open is committed to open peer review. As part of this commitment we make the peer review history of every article we publish publicly available.

When an article is published we post the peer reviewers' comments and the authors' responses online. We also post the versions of the paper that were used during peer review. These are the versions that the peer review comments apply to.

The versions of the paper that follow are the versions that were submitted during the peer review process. They are not the versions of record or the final published versions. They should not be cited or distributed as the published version of this manuscript.

BMJ Open is an open access journal and the full, final, typeset and author-corrected version of record of the manuscript is available on our site with no access controls, subscription charges or pay-per-view fees (http://bmjopen.bmj.com).

If you have any questions on BMJ Open's open peer review process please email editorial.bmjopen@bmj.com

BMJ Open

Generalized anxiety disorder and health service use: findings from a large, population study

Journal:	*BMJ Open*
Manuscript ID	bmjopen-2017-018539
Article Type:	Research
Date Submitted by the Author:	05-Jul-2017

Complete List of Authors:	Remes, Olivia; University of Cambridge, Public Health and Primary Care<br>Wainwright, Nicholas; University of Cambridge, UK, Public Health and Primary Care (retired)<br>Surtees, Paul; University of Cambridge, Department of Public Health and Primary Care<br>LaFortune, Louise; University of Cambridge, Institute of Public Health<br>Khaw, Kay-Tee; University of Cambridge, Department of Public Health and Primary Care<br>Brayne, Carol; University of Cambridge, Institute of Public Health

**Primary Subject Heading**:	Mental health
Secondary Subject Heading:	Health services research, Public health
Keywords:	EPIDEMIOLOGY, MENTAL HEALTH, PUBLIC HEALTH

SCHOLARONE™ Manuscripts

For peer review only

For peer review only - http://bmjopen.bmj.com/site/about/guidelines.xhtml

Generalized anxiety disorder and health service use: findings from a large, population study

Olivia Remes[1], Nick Wainwright[2], Paul Surtees[1], Louise Lafortune[1], Kay-Tee Khaw[3], Carol Brayne[1]

[1]Department of Public Health and Primary Care, University of Cambridge, Forvie Site, Robinson Way, Cambridge, UK CB2 0SR

[2](Retired) Department of Public Health and Primary Care, University of Cambridge, 2 Worts' Causeway, Cambridge, UK CB1 8RN

[3]Department of Public Health and Primary Care, University of Cambridge, 2 Worts' Causeway, Cambridge, UK CB1 8RN

Figures: 1; Tables: 3; Word count: 3783

Correspondence to: Olivia Remes or260@medschl.cam.ac.uk, telephone: 07776740550

For peer review only

ABSTRACT

OBJECTIVE

Generalized anxiety disorder is the most common anxiety disorder in the general population, and has been associated with high economic and human burden. However, it has been neglected in the health services literature, with the exception of some studies showing that it contributes to higher use of primary care services. The objective of this study will be to assess whether generalized anxiety disorder leads to hospital admissions using data from the European Prospective Investigation of Cancer-Norfolk.

DESIGN

Large, population study.

SETTING

UK population-based cohort.

PARTICIPANTS

30,445 people over the age of 40 were recruited through general practice registers in England. Of these, 21,000 completed a structured health and lifestyle questionnaire used to assess generalised anxiety disorder. Anxiety was examined in 1996-2000, and health service use was captured between 1999/00 and 2009 through record linkage with large, administrative health databases. 18,076 participants had complete data on covariates.

MAIN OUTCOME MEASURE

Past-year generalised anxiety disorder defined according to the Diagnostic and Statistical Manual of Mental Disorders, fourth edition.

RESULTS

In this study, 2.2% (397/18,076) respondents had generalised anxiety disorder. Anxiety was not independently associated with hospital admissions (IRR=1.01, 95% CI: 0.87, 1.16) over nine years. However, those who developed anxiety before 30 years of age were at high risk for hospital service use (IRR=1.24, 95% CI: 1.02, 1.50).

CONCLUSION

People with an earlier age of onset for generalized anxiety disorder had a higher risk for hospital admissions over 9 years between 1999/00 and 2009 in the European Prospective Investigation of Cancer-Norfolk study. Altogether, our findings show that anxiety that developed early in life may be of a more severe form, and is associated with high health service use rates. People with early-onset anxiety might be in poorer health than others; clinicians should thus consider taking psychiatric histories of patients presenting with anxiety complaints.

Key words: Anxiety, anxiety disorders, health services

For peer review only

ARTICLE SUMMARY

Strengths and limitations of this study

- We used a large, population-based sample of middle- and older-aged adults and adjusted for a range of important confounders, such as, sociodemographic factors and medical history.
- We used a structured, self-reported questionnaire to assess presence of past-year GAD, and participants were followed for 9 years.
- We examined health services through record linkage with large, administrative health databases.
- Those who participated in this study were somewhat less deprived and healthier than individuals living in other parts of England; therefore, our results may not generalize to people living in extremely deprived circumstances.

For peer review only

INTRODUCTION

Anxiety disorders[1] are the most common class of psychiatric disorders in the general population. The Global Burden of Disease study[2] estimated that anxiety disorders contribute to 26.8 million disability adjusted life years, and their annual direct cost is $42.3 billion[3]. Generalized anxiety disorder (GAD) is a prevalent and disabling condition in adults, and can lead to serious impairment in social and occupational functioning.[4] It is associated with poor quality of life, impaired functioning and risk of suicide.[5-8] Across the anxiety disorders, GAD has been found to be the most debilitating.[5, 9] Although there is effective treatment for GAD, only a third of those affected receive any treatment.[8] This is because anxiety disorders are frequently under-recognized and mismanaged by clinicians in primary care, which is often the first point of contact for those with mental health problems.

Previous literature has suggested that patients with mental health problems tend to present to their physician with physical rather than psychiatric symptoms, which leads to the administration of extensive and costly medical tests in the search for a physical cause. This implies that the problem has no physical basis (it is purely psychiatric in nature), and that the patient, failing to recognize this, continues frequenting the general medical setting until the underlying problem has been resolved.[9] Studies reiterate that effective treatment for anxiety exists, but because physicians fail to recognize the origin of the patient's complaints as psychiatric rather than physical, patients are not given the anxiolytic medication or psychotherapy needed, hindering their recovery. However, it might be that anxiety represents more than just worry-related symptoms that cannot be simply resolved through psychological therapies or psychotropic medication. Anxiety disorders could be masking underlying poor health or could be an early warning signal for future health problems that are not yet detectable by standard medical tests.

Anxiety has been linked to hypothalamic-pituitary-adrenal (HPA)-axis dysregulation, inflammation, and the release of pro-inflammatory cytokines, and this can lead to poor health.[9] A recent study of hospitalized patients[10] also showed that people with anxiety disorders had more physical comorbidities, including cardiovascular diseases and their risk factors, compared to people without anxiety disorders.

For peer review only

Early or late-onset forms of anxiety disorders should be also considered. A study[11] of over one million Swedish men followed for over 20 years showed that early-onset forms of mental disorders in particular led to increased risk of incident CHD. Therefore, identifying clinical aspects, such as, individual anxiety disorders, early or late onset forms of the disorder, and episode chronicity and frequency, can lead to better clinical management and more accurate prediction of future disability and health service use.[12]

The evidence base on health service use and anxiety is small and limited, and most of the recent studies have focussed on post-traumatic stress disorder (PTSD) in war veterans.[13, 14] Most studies are based on clinical populations, cross-sectional designs, small samples, short follow-up periods (usually one year), do not adequately control for confounders, and use self-report to assess frequency or other measures of hospitalization patterns in the clinical setting.

Generalized anxiety disorder (GAD) is one of the most common anxiety disorders in the general population[15] and the primary care setting[16], and has been associated with high economic and human burden. However, it has been neglected in the health services literature, with the exception of some studies showing GAD to contribute to higher use of primary care services in primary care samples.[17-20] Whether GAD leads to hospital admissions is unknown.

The objective of this study will be to assess the association between generalized anxiety disorder (GAD) and hospital service use in a longitudinal, population cohort of over 18,000 British individuals followed for 10 years. The aim is also to determine whether early or late onset forms of the disorder, episode frequency and chronicity contribute to higher rates of hospital services.

For peer review only

METHODS

Study population

The study population was drawn from the European Prospective Investigation of Cancer-Norfolk longitudinal, cohort study, described in detail elsewhere.[21, 22] Briefly, a total of 30,445 participants over the age of 40 living in Norwich and the surrounding towns and rural areas were recruited between 1993 and 1997 using general practice registers. At baseline, they completed a health questionnaire capturing sociodemographics and medical history, and underwent a health check that gathered information on medication use. During follow-up, between 1993 and 1999/2000, participants completed self-reported postal questionnaires provided they: 1) were still alive, 2) did not ask to be removed from the study's mailing list, and 3) had a valid mailing address. Between 1996 and 1999/2000, respondents completed a Health and Life Experiences Questionnaire (HLEQ) used to capture information on psychiatric disorders, other psychosocial factors, and risk behaviours. Record linkage with administrative health databases using a unique identifier was used to determine hospitalization admissions data until 2009.

All participants recruited through general-practice registers and who completed a baseline health questionnaire were eligible to be included in our study; those who completed a psychosocial questionnaire during follow-up were eligible to be included in our analysis.

Assessment of generalized anxiety disorder (GAD)

The HLEQ was used to derive a measure of GAD according to the Diagnostic and Statistical Manual of Mental Disorders, fourth edition. The HLEQ captured the onset and offset timings of episodes of past-year GAD.[23] Past-year GAD consisted of at least one episode that had offset within 12 months of administration of the HLEQ. DSM-IV GAD was diagnosed if participants reported having uncontrollable, excessive worry for six months or longer on most days than not that resulted in disability or impairment. In addition, at least three of the following symptoms needed to have been present: restlessness, irritability, muscle

For peer review only - http://bmjopen.bmj.com/site/about/guidelines.xhtml

tension, fatigue, trouble concentrating because of worry, mind going blank, trouble falling asleep, trouble staying asleep, and feeling keyed up or on edge.

Assessment of covariates (potential confounders)

Potential confounders (based on the literature) included sociodemographics (age, sex, education, marital status, social class, employment), prevalent physical diseases, MDD, psychotropic medication use (antihypnotics and antidepressants), and risk behaviours (alcohol use, smoking, physical activity). The final categorization of the variables took cell size into account and was also done in accordance with previous literature.[23, 24-29] Age was first assessed as a categorical variable, and subsequently divided into 10-year bands. Sex was categorized into male vs. female; marital status was categorized into: married, single (or never married), and others (widowed, divorced, separated); educational attainment into high (vocational or formal qualifications at the A- or O-level or degree-level qualifications) vs. low (no formal qualifications). Social class was derived using the Computer-Assisted Standard Occupational Coding[29] and categorized as follows: I (professionals), II (managerial and technical occupations), III non-manual and III manual (skilled workers), IV (partly skilled workers), and V (unskilled manual workers). To assign social class to men and women, the male partner's current or past occupation was used. If this information was not available, the female partner's occupation was used. If the social class from either partner was unavailable, then it was coded as missing. The final categorization of social class included manual: skilled manual, partly skilled, and unskilled; and non-manual: professionals, managerial and technical, and skilled non-manual. Marital status was categorized into three groups: married, single (or never married), and others (widowed, divorced, separated). Employment was divided into yes vs. no. Behaviour risk factor measures included alcohol intake (units of alcohol/week), smoking status (current, former, non-smoker), and physical activity (inactive, moderately inactive, moderately active, active). Use of medications included hypnotic drug use (yes/no) and antidepressant drug use (yes/no). Presence of past-year DSM-IV major depressive disorder (MDD) (yes/no) was also assessed.[30]

For peer review only

Individual-level health status was assessed through the construction of a variable capturing major prevalent physical diseases associated with anxiety.[31] This was based on HLQ questions asking participants: "Has the doctor ever told you that you have any of the following?", followed by a list of options, such as stroke, myocardial infarction, and cancer. To determine disability levels, we used the physical component summary score (PCS) of the Medical Outcomes Study 36-Item Short Form (SF-36), a widely-used, validated self-assessment tool. A score of 100 represents no disability and 0 represents a high level of disability.[32] PCS scores were dichotomized above and below the median.

All of these individual-level variables were regarded as potential confounders and selected based on the literature and their association with anxiety and deprivation.[33, 34]

Hospital service use

All analyses are based on non-psychiatric hospitalizations. Primary care service use was not captured in this study.

Frequency of hospitalization between 1999/00 and 2009 was determined using administrative health databases maintained by the National Health Service. The East Norfolk Primary Health Care trust databases were used, and these are updated on an ongoing basis and provide information on clinical and administrative data from participating facilities, such as, hospitals.

England is under a publicly-funded health care system (the National Health Service), free at the point of delivery; therefore, we expect factors, such as access to health insurance or personal income, to have minimal impact on the care that is obtained by study participants. The databases used in this study are maintained by the National Health Service, which is likely to capture most hospital admissions from the population, as private sector provision is minimal. This means that admissions data in our study can be considered complete for the ascertainment of hospital/health service use, and the likelihood of bias minimal. To access hospital services in the UK, a referral is needed from the primary care practitioner, who acts as a gate-keeper to secondary care.

For peer review only

The East Norfolk Primary Health Care databases were linked to the EPIC-Norfolk cohort using participants' unique National Health Service number, which allows complete record linkage across settings and calendar time.

Vital status for participants was determined through record linkage with the United Kingdom Office of National Statistics. Vital status was available for all participants. This allowed us to exclude those who died before their health service use was ascertained.

Statistical Analysis

First, demographics, social class, medical and psychiatric co-morbidities, risk behaviours, and medication use were compared by GAD status. Second, frequency of hospitalization was compared by GAD status.

Since the number of hospital admissions was skewed and the variance was much larger than the mean, zero-inflated negative binomial regression was used for frequency of hospital utilization (number of hospital admissions). The log-likelihood test showed that this model was superior to Poisson regression. Three models were fitted for hospital admissions with progressive adjustment of covariates: model A adjusted for sociodemographics (age, sex, education, social class, employment), physical comorbidities and disability; model B further accounted for past-year MDD (assessed at the same questionnaire point as past-year GAD); and model C further controlled for antihypnotic and antidepressant medications, physical activity, alcohol, and smoking.

Finally, we determined whether the risk for hospitalization was higher among those with: 1) 3 or more episodes of lifetime GAD (versus fewer than 3 episodes), 2) age of onset at 30 years or younger (versus over 30 years), and 3) episodes that lasted on average 6 months or more (versus fewer than 6 months). Two-sided statistical tests were conducted and a p-value of <0.05 was used for statistical significance. Analyses were implemented in SAS, version 9.3.

For peer review only

To arrive at the study size, we went through the following steps: of the 30,445 who completed the baseline HLQ, we retained those participants who completed the HLEQ (20,921), and of these, we kept those people with complete data on all covariates (18,076). (Figure 1)

Patient involvement

There were no patients involved in the development of the research question and outcome measures, the design of the study, or the recruitment to and conduct of the study.

For peer review only

RESULTS

Of the 30,445 people recruited at baseline, 20,921 participants completed the HLEQ; we had 479 missing observations for past-year GAD, 700 for past-year MDD, 1386 for SF-36, and the rest of the missing observations (280) were generated from the other covariates. The final sample included a total of 18,076 participants. Participants were assessed between 1999/00 and 2009 (followed for 9 years) (Figure 1).

In 1996-2000, GAD was present in 397 out of 18,076 (2.2%) people. Table 1 shows the baseline characteristics of participants by GAD status.

For peer review only

Table 1 Percentage and number of people with past-year GAD reported in 1996-2000 according to sociodemographic factors, health status, and behaviour risk factors for the EPIC-Norfolk cohort (n=18,076)

Characteristic	Number with characteristic	Percentage and number with past-year GAD
**Socio-demographics**		
**Age (years)**		
<50	2369	3.4 (81)
50-60	6242	2.9 (180)
60-70	5782	1.6 (94)
70+	3683	1.1 (42)
**Sex**		
Women	10037	2.5 (252)
Men	8039	1.8 (145)
**Education**[‡]		
Low	6189	2.0 (122)
High	11887	2.3 (275)
**Marital status**		
Single	690	3.6 (25)
Married	14640	2.0 (287)
Other[*]	2746	3.1 (85)
**Social class**		
Manual	6910	2.0 (139)
Non-manual	11166	2.3 (258)
**Employment**		
Yes	7742	2.0 (156)
No	10334	2.3 (241)
**Health status**		
**Physical comorbidities**[+]		
Yes	9237	2.7 (253)
No	8839	1.6 (144)
**Disability level**		
High[¶]	8989	3.0 (270)
Low	9087	1.4 (127)
**Psychiatric comorbidity**		
**Past-year MDD**		
Yes	941	21.6 (203)
No	17135	1.1 (194)
**Lifestyle**		
**Physical activity**		
Active[¥]	12882	2.1 (272)
Inactive	5194	2.4 (125)
**Smoking status**		
Current smoker	1907	4.8 (92)
Former smoker	7510	1.9 (141)
Never smoker	8659	1.9 (164)
**Alcohol intake**		
High[α]	9242	2.0 (183)

For peer review only

Low	8834	2.4 (214)
**Medication**		
**Antidepressant use**		
Yes	632	15.4 (97)
No	17444	1.7 (300)
**Antihypnotic use**		
Yes	2799	2.4 (68)
No	15277	2.2 (329)

[+] Prevalent physical disease: respiratory disease (asthma and bronchitis), allergies and hay fever, stroke, heart attack, cancer, diabetes, thyroid conditions, arthritis

[¥] Moderately inactive, moderately active, active

[‡] High education: O-level, A-level, degree; low education: refers to no education

[*] Other: divorced, separated, widowed

[¶] Below the median PCS value of 50.6

[α] 3+ units of alc./week (1 pint beer=2 units, 1 glass wine=1 unit, 1 glass sherry=1 unit, 1 glass spirits=1 unit)

For peer review only

317 Those with GAD were more likely to be younger, women, inactive, current smokers, low
318 alcohol consumers, of higher educational attainment, single, non-manual social class,
319 without employment, with physical comorbidities, high levels of disability, MDD, and to take
320 antidepressant and antihypnotic medications. Table 2 summarizes the means and standard
321 deviations of the number of hospital admissions by participant characteristics.
322

For peer review only

Table 2: Hospital admissions (mean, SD) by participant characteristics in 18,076 British people between 1999/00 and 2009

	Total number with characteristic	Number of admissions
**Characteristic**		**Mean (SD)**
**Past-year GAD**		
Yes	397	4.1 (6.3)
No	17679	3.4 (13.0)
**Socio-demographics**		
**Age (years)**		
<50	2369	1.9 (9.8)
50-60	6242	3.0 (16.4)
60-70	5782	3.8 (11.1)
70+	3683	4.6 (9.6)
**Sex**		
Women	10037	3.1 (14.0)
Men	8039	3.9 (11.3)
**Education**[‡]		
Low	6189	4.1 (17.0)
High	11887	3.1 (10.0)
**Marital status**		
Single	690	3.0 (8.9)
Married	14640	3.4 (10.9)
Other[*]	2746	4.0 (20.9)
**Social class**		
Manual	6910	4.0 (18.2)
Non-manual	11166	3.1 (7.9)
**Employment**		
Yes	7742	2.5 (9.1)
No	10334	4.1 (15.0)
**Health status**		
**Physical comorbidities**[+]		
Yes	9237	3.9 (10.4)
No	8839	3.0 (15.0)
**Disability level**		
High[¶]	8989	4.4 (16.4)
Low	9087	2.5 (7.8)
**Psychiatric comorbidity**		
**Past-year MDD**		
Yes	941	4.6 (13.6)
No	17135	3.4 (12.8)
**Lifestyle**		
**Physical activity**		
Active[¥]	12882	3.2 (13.3)
Inactive	5194	4.1 (11.6)
**Smoking status**		
Current smoker	1907	4.6 (26.7)

For peer review only

Former smoker	7510	3.8 (11.4)
Never smoker	8659	2.9 (8.9)
**Alcohol intake**		
High[α]	9242	3.2 (13.3)
Low	8834	3.7 (12.4)
**Medication**		
**Antidepressant use**		
Yes	632	5.2 (19.8)
No	17444	3.4 (12.5)
**Antihypnotic use**		
Yes	2799	5.2 (23.6)
No	15277	3.1 (9.6)

[+] Prevalent physical disease: respiratory disease (asthma and bronchitis), allergies and hay fever, stroke, heart attack, cancer, diabetes, thyroid conditions, arthritis

[¥] Moderately inactive, moderately active, active

[‡] High education: O-level, A-level, degree; low education: refers to no education

[*] Other: divorced, separated, widowed

[¶] Below the median PCS value of 50.6

[α] 3+ units of alc./week (1 pint beer=2 units, 1 glass wine=1 unit, 1 glass sherry=1 unit, 1 glass spirits=1 unit)

For peer review only

Participants with GAD had a higher frequency of hospitalization compared to those without GAD. Some of the findings show that frequency of hospitalization was markedly higher among older age groups, men, those with low educational attainment, unemployed participants, those with high levels of disability, past-year MDD and individuals taking medication.

Table 3 shows the unadjusted and adjusted incidence rate ratios of hospital admissions by GAD status.

For peer review only

Table 3: Associations between past-year GAD reported in 1996-2000 and hospital admissions in 1999/00-2009 in 18,076 British people over the age of 40

IRR and 95% CI				
**Characteristic**	**Crude IRR**	**A[1]**	**B[2]**	**C[3]**
**Past-year GAD**				
Yes	1.19 (1.03, 1.37)	1.26 (1.10, 1.45)	1.11 (0.96, 1.28)	1.01 (0.87, 1.16)
No	1.00	1.00	1.00	1.00
**Socio-demographics**				
**Age**				
Per 10 years	1.36 (1.33, 1.39)	1.19 (1.16, 1.23)	1.20 (1.17, 1.24)	1.20 (1.16, 1.24)
**Sex**				
Women	0.80 (0.77, 0.83)	0.76 (0.73, 0.79)	0.76 (0.72, 0.79)	0.77 (0.73, 0.80)
Men	1.00	1.00	1.00	1.00
**Education[‡]**				
Low	1.30 (1.25, 1.36)	1.13 (1.08, 1.18)	1.14 (1.09, 1.19)	1.12 (1.07, 1.17)
High	1.00	1.00	1.00	1.00
**Marital status**				
Single	0.88 (0.79, 0.99)	0.85 (0.77, 0.95)	0.85 (0.76, 0.94)	0.84 (0.75, 0.93)
Married	1.00	1.00	1.00	1.00
Other[*]	1.21 (1.14, 1.28)	1.17 (1.10, 1.24)	1.14 (1.07, 1.20)	1.08 (1.02, 1.14)
**Social class**				
Manual	1.29 (1.24, 1.35)	1.25 (1.19, 1.30)	1.25 (1.19, 1.30)	1.19 (1.14, 1.25)
Non-manual	1.00	1.00	1.00	1.00
**Employment**				
Yes	1.00	1.00	1.00	1.00
No	1.63 (1.57, 1.70)	1.18 (1.12, 1.25)	1.17 (1.11, 1.24)	1.13 (1.08, 1.20)
**Health status**				
**Physical comorbidities[+]**				
Yes	1.32 (1.27, 1.38)	1.18 (1.13, 1.23)	1.17 (1.13, 1.23)	1.17 (1.12, 1.22)
No	1.00	1.00	1.00	1.00
**Disability level**				
High[¶]	1.78 (1.71, 1.85)	1.51 (1.45, 1.58)	1.51 (1.44, 1.57)	1.43 (1.37, 1.50)
Low	1.00	1.00	1.00	1.00
**Psychiatric comorbidity**				
**Past-year MDD**				
Yes	1.35 (1.23, 1.48)		1.34 (1.22, 1.48)	1.29 (1.18, 1.42)
No	1.00		1.00	1.00
**Medications**				
**Antihypnotic use**				
Yes	1.67 (1.58, 1.77)			1.28 (1.21, 1.35)
No	1.00			1.00
**Antidepressant use**				
Yes	1.54 (1.38, 1.72)			1.27 (1.14, 1.42)

For peer review only

No	1.00			1.00
**Lifestyle**				
**Physical activity**				
Active[¥]	1.00			1.00
Inactive	1.27 (1.21, 1.33)			1.04 (0.99, 1.08)
**Smoking status**				
Current smoker	1.59 (1.48, 1.71)			1.47 (1.37, 1.57)
Former smoker	1.32 (1.26, 1.38)			1.12 (1.07, 1.17)
Never smoker	1.00			1.00
**Alcohol intake**				
High[α]	0.88 (0.85, 0.92)			0.92 (0.88, 0.96)
Low	1.00			1.00

[1] Model A: adjusted for age, sex, education, marital status, social class, employment, physical comorbidities

[2] Model B: adjusted for sociodemographics, physical comorbidities, MDD

[3] Model C: adjusted for sociodemographics, physical comorbidities, MDD, antihypnotic use, antidepressant use, physical activity, smoking, alcohol

[+] Physical co-morbidities: respiratory disease (asthma and bronchitis), allergies and hay fever, stroke, heart attack, cancer, diabetes, thyroid conditions, arthritis

[¥] Moderately inactive, moderately active, active

[‡] High education: O-level, A-level, degree; low education: refers to no education

[*] Other: divorced, separated, widowed

[¶] Below the median PCS value of 50.6

[α] 3+ units of alc./week (1 pint beer=2 units, 1 glass wine=1 unit, 1 glass sherry=1 unit, 1 glass spirits=1 unit)

For peer review only

After adjustment for sociodemographic variables, physical comorbidities, and disability, GAD was associated with a 26% higher incidence rate of hospitalization (IRR=1.26, 95% CI: 1.10, 1.45). The incidence rate ratio was somewhat attenuated and became statistically non-significant after further adjustment for MDD (IRR=1.11, 95% CI 0.96, 1.28). The effect estimate approached the null after further adjustment for medication use and lifestyle factors (IRR=1.01, 95% CI: 0.87, 1.16).

Next, we assessed whether risk for hospital admissions varied by frequency of lifetime episodes, age of onset, and episode chronicity. People who developed GAD before 30 years of age were 24% more likely to be admitted to the hospital than those who developed it later in life (IRR=1.24, 95% CI: 1.02, 1.50). People with more than 3 lifetime episodes also had a higher risk of hospitalizations, but the association did not reach statistical significance (IRR=1.15, 95% CI: 0.98, 1.35). Those whose episodes lasted, on average, 6 months or longer did not have an increased risk for admissions compared to those with shorter episodes (IRR=1.09, 95% CI: 0.87, 1.36).

For peer review only

DISCUSSION

This is the first study to assess the association between generalized anxiety disorder (GAD) and hospital service use in a population-based cohort. This longitudinal study showed that having an episode of GAD in the past year was not independently associated with hospital admissions during the subsequent ten years. However, those who developed GAD at an early age (before 30) were at a significantly increased risk for being admitted to hospital than those with a later onset.

People with past-year GAD were more likely to have medical comorbidities; therefore, the association with hospital service use was partially driven by worse physical health in those with anxiety. Psychiatric co-morbidity also led to attenuation of the effect estimate for those who experienced an episode of GAD in the past year. Major depressive disorder (MDD) is highly comorbid with GAD, and when it was introduced in the models, it explained away any remaining associations with hospital service utilization in the primary analyses.

When the course of GAD was considered, the risk for hospitalization was found to be much higher in those who developed anxiety at an early age, after adjusting for physical diseases, psychiatric comorbidity, and behaviour risk factors.

Strengths and limitations

There are several strengths associated with our study. We had a large, population-based sample of middle- and older-aged adults and adequately adjusted for a range of possible confounders. We used a structured questionnaire to assess past-year GAD according to DSM-IV criteria, used large administrative health databases to examine hospital service use (avoiding the self-reporting bias found in questionnaire studies), and participants were followed for a long time. We had a large list of self-reported physician diagnoses of chronic diseases that we used to ascertain medical histories. Despite this, the residual effect of diseases not captured by our study, but that are associated with GAD may be present. Past illness may have been underreported, which may have introduced measurement error and further attenuated effect estimates towards the null. A negligible proportion of participants

For peer review only

may have obtained care at private facilities, which might have led to non-differential misclassification. The databases used in this study also did not capture admissions to hospitals outside the UK. However, migration in the EPIC-Norfolk cohort is minimal and does not present a problem.

We may have overadjusted our models with the inclusion of self-evaluated impairment, as this may be part of the expression of psychiatric illness. This can lead to attenuation of effect estimates. If participants chose not to answer certain questions in the HLEQ, this contributed to missing data; however, to avoid biasing the findings, we retained participants for whom we had complete data on all covariates. Non-participation in our study also may have led to non-differential misclassification and further attenuation of effect estimates.

Another limitation is that we did not have data on primary care service use. Merging population cohorts, such as ours, with primary care service administrative databases and hospitalization databases would have provided a more complete picture of the burden of GAD on the health care system.

Participants were required to complete detailed dietary and lifestyle questionnaires and undergo periodic health assessments. Because those who participated in EPIC-Norfolk were more affluent and healthier than individuals living in other parts of England, our results may not generalize to people living in extremely deprived areas.

Comparison with other studies

Most of the studies assessing the link between psychiatric disorders and non-psychiatric health service utilization have focused on depression and, to a lesser extent, panic disorder and PTSD, while other anxiety disorders have been significantly underresearched. Most of the studies on depression have shown an association with health service use in both clinical and community samples.[35] There are substantially fewer studies on anxiety, and a number of these have shown positive associations with health service use. A US study[36] that recruited patients from an outpatient clinic showed that anxiety disorders were linked to higher utilization of primary care services compared to depressive or addictive disorders.

For peer review only

Patients, however, were recruited from an outpatient clinic located in a predominantly rural area, which might have affected generalizability. Another study showed anxiety disorders to be associated with a higher number of consultations in general medical, emergency and specialty settings, such as cardiology and dermatology.[37] In this study, people were sampled from an anxiety clinic, thereby leading to possible selection bias. PTSD has also been associated with health service use, such as more ordered lab tests and medications prescribed compared to those without this disorder, but much of the literature on this[14, 38] has been based on highly-select samples that have limited generalizability. Two of the more recent studies on GAD showed it to be associated with health care use. One Canadian study[19] suggested a higher rate of medical visits to primary care practitioners in those with GAD, while a US study[17] also found a higher frequency of specialty medical care visits in affected individuals. Both of these studies recruited clinical samples, with the potential for self-selection bias. None of these studies assessed whether the severity of anxiety contributes to even higher health service use rates (early onset forms of the disorder are typically the most severe, hardest to treat and with the poorest prognosis [9]).

Mechanisms

A more severe course of GAD, marked by early age of onset, can lead to higher rates of health services because of unhealthy behaviours, such as smoking and alcohol. When we controlled for these covariates, the associations with hospitalizations remained significant. It could also be that anxiety is associated with poorer underlying health, which leads to higher health service use rates. Although we controlled for several chronic diseases, we might have missed some conditions that are associated with GAD and hospitalizations. A third explanation for higher health service use in those with anxiety could relate to inflammatory pathways. If clinically apparent signs of disease have not yet developed in those with anxiety or are at an early, undetectable stage, it will not be possible to measure these factors and adjust for them in analyses.

For peer review only

Implications

Generalized anxiety disorder is a debilitating and impairing condition.[9] The evidence base on its association with health services is small and confined to clinical settings with the potential for self-selection bias. Our study overcomes many limitations of previous studies, and shows, for the first time that early age of onset of generalized anxiety disorder is associated with increased use of hospital services in adults, after controlling for a range of important confounders.

Population-based research on anxiety is lacking, and thus far, no studies have assessed the association between GAD and non-psychiatric hospitalization. Clinicians should consider that it is not just the diagnosis of the disorder at one point in time that is predictive of deleterious health outcomes; its long-term course is also important. GAD has a waxing and waning course throughout a patient's life, and many of those affected experience relapse after psychiatric treatment. Furthermore, early-onset forms of the disorder have been shown to be more severe, and are the most difficult to treat and have a poor prognosis.[9] As such, examining the course of the disorder and determining the age of onset makes theoretical sense and has clinical implications. Taking a patient's medical history when the presenting complaints are related to anxiety can differentiate those with milder forms (knowing this can reduce unnecessary follow-up investigations) from those with more severe cases that would benefit from medical follow-up. Assessing the presence of GAD using DSM-IV criteria is time-consuming in a busy, clinical practice; hence, future studies should replicate our study using brief, validated screening tools. Our findings are also important for policy-makers. Large numbers of people are affected by anxiety and develop GAD early in life (this group then shows a disproportionate consumption of health services)[9], therefore, policy-makers should consider rolling out more widespread anxiety prevention and screening programmes.

We would like to make some recommendations for future research. Younger people are most affected by anxiety disorders[31], therefore, future studies should assess these associations in younger cohorts. Second, clinical studies have shown depression to be highly co-morbid with GAD[16], hence, the impact of comorbidity on the health care system

For peer review only

should also be examined. Third, previous studies suggest, that across the anxiety disorders, GAD is the most likely to lead to the development and maintenance of chronic conditions.[9] To this end, the mediating effect of chronic conditions in relation to anxiety should be examined. Finally, future research should merge a population-based cohort with primary and secondary care administrative health databases to provide a more complete picture of the burden of anxiety on the health care system.

Conclusion

People with an earlier age of onset for generalized anxiety disorder (GAD) had a higher risk for hospital admissions over 10 years between 1996-1999 and 2009 in the European Prospective Investigation of Cancer in Norfolk study. Altogether, our findings show that a more severe course of anxiety is associated with high health service use rates.

For peer review only

Acknowledgements: OR received a PhD studentship from the National Institute for Health Research.

Competing interest: All authors have completed the ICMJE uniform disclosure form at www.icmje.org/coi_disclosure.pdf and declare: no support from any organisation for the submitted work; no financial relationships with any organizations that might have an interest in the submitted work in the previous three years; no other relationships or activities that could appear to have influenced the submitted work.

Funding: This work was supported by the Medical Research Council UK (grant number SP2024-0201 and SP2024-0204) and Cancer Research UK (grant number G9502233).

Author contributions: OR (corresponding author) had the idea for and conducted the analysis, and wrote the article. CB critically reviewed drafts of the manuscript, KK edited versions of the paper; PS and NW provided feedback into the analysis. OR, CB, KK, LL, PS, and NW contributed to the interpretation of data for the work, agreed to be accountable for all aspects of the work, gave final approval of the version to be published, and made substantial contributions to the analysis and interpretation of data. OR, CB, KK, LL, PS, and NW have seen and approved the final version. OR, CB, KK, LL, PS, and NW had full access to all the data in the study and take responsibility for the integrity of the data and the accuracy of the data analysis. OR acts as guarantor of the study.

Transparency declaration: OR affirms that the manuscript is an honest, accurate, and transparent account of the study being reported; that no important aspects of the study have been omitted; and that any discrepancies from the study as planned have been explained.

Role of study sponsors and statement of independence: The funding sources had no role in the design and conduct of the study; collection, management, analysis, and interpretation of the data; and preparation, review, or approval of the manuscript.

For peer review only

Ethical approval: The study has ethics committee approval from Norfolk Ethics Committee (Rec Ref: 98CN01) and all participants gave informed consent.

Data sharing: No additional data available. Original dataset requests should be sent to the corresponding author. Please contact O Remes at or260@medschl.cam.ac.uk for questions about the statistical code.

Copyright: "The Corresponding Author has the right to grant on behalf of all authors and does grant on behalf of all authors, a worldwide licence to the Publishers and its licensees in perpetuity, in all forms, formats and media (whether known now or created in the future), to i) publish, reproduce, distribute, display and store the Contribution, ii) translate the Contribution into other languages, create adaptations, reprints, include within collections and create summaries, extracts and/or, abstracts of the Contribution, iii) create any other derivative work(s) based on the Contribution, iv) to exploit all subsidiary rights in the Contribution, v) the inclusion of electronic links from the Contribution to third party material where-ever it may be located; and, vi) licence any third party to do any or all of the above."

For peer review only

REFERENCES

1. American Psychiatric Association. (1994). *Diagnostic and Statistical Manual of Mental Disorders* (4th edn). Washington, DC: APA.
2. Whiteford HA, Degenhardt, Rehm J, et al. Global burden of disease attributable to mental and substance use disorders: findings from the global burden of disease study 2010. *Lancet* 2013;13:1575-86 doi: http://dx.doi.org/10.1016/S0140-6736(13)61611-6.
3. Greenberg PE, Sisitsky T, Kessler RC, et al. The economic burden of anxiety disorders in the 1990s. *J Clin Psychiatry* 1999;60:427-35.
4. Kroenke K, Spitzer RL, Williams JB, et al. Anxiety disorders in primary care: prevalence, impairment, comorbidity, and detection. *Ann Intern Med* 2007;146:317-25 doi: 10.7326/0003-4819-146-5-200703060-00004.
5. Comer JS, Blanco C, Hasin DS, et al. Health-related quality of life across the anxiety disorders. *J Clin Psychiatry* 2011;72:43–50 doi: 10.4088/JCP.09m05094blu [published Online First 24 August 2010].
6. Tyrer P, Baldwin D. Generalised anxiety disorder. *Lancet* 2006;16:2156–66 doi: http://dx.doi.org/10.1016/S0140-6736(06)69865-6.
7. Hoge E, Ivkovic A, Fricchione G. Generalized anxiety disorder: diagnosis and treatment. *BMJ* 2012;345:e7500 doi: https://doi.org/10.1136/bmj.e7500.
8. Revicki DA, Travers K, Wyrwich KW. Humanistic and economic burden of generalized anxiety disorder in North America and Europe. *J Affect Disord* 2012;140:103–12 doi: 10.1016/j.jad.2011.11.014 [published Online First 9 December 2011].
9. Simpson HB, Neria Y, Lewis-Fernandez R, et al. Anxiety disorders. Theory, research and clinical perspectives. New York, US: Cambridge University Press 2010.

For peer review only

10. Schoepf D, Heun R. Anxiety disorders and physical comorbidity: increased prevalence but reduced relevance of specific risk factors for hospital-based mortality during a 12.5-year observation period in general hospital admissions. *Eur Arch Psychiatry Clin Neurosci* 2015;265:387-98 doi: 10.1007/s00406-014-0566-9 [published Online First 4 December 2014].

11. Gale CR, Batty GD, Osborn DP, et al. Mental disorders across the adult life course and future coronary heart disease: evidence for general susceptibility. *Circulation* 2014;129:186-93 doi: 10.1161/CIRCULATIONAHA [published Online First 4 November 2013].

12. Maj M. ‘Psychiatric comorbidity’: an artefact of current diagnostic systems. *Br J Psychiatry* 2005;186:182-84 doi: 10.1192/bjp.186.3.182 [published Online First 28 February 2005].

13. Bryan CJ, Stephenson JA, Morrow CE, et al. Posttraumatic stress symptoms and work-related accomplishment as predictors of general health and medical utilization among special operations forces personnel. *J Nerv Ment Dis* 2014;202:105–10. doi: 10.1097/NMD.0000000000000076.

14. Lehavot K, Der-Martirosian C, Simpson TL, et al. The role of military social support in understanding the relationship between PTSD, physical health, and healthcare utilization in women veterans. *J Trauma Stress*. 2013;26:772–5 doi: 10.1002/jts.21859 [published Online First 6 November 2013].

15. Stein MB. Public health perspectives on generalized anxiety disorder. *J Clin Psychiatry* 2004;65:3-7.

For peer review only

16. Nutt D, Argyropoulos S, Hood S, et al. Generalized anxiety disorder: a comorbid disease. *Eur Neuropsychopharmacol* 2006;16:S109-18 doi: 10.1016/j.euroneuro.2006.04.003 [published Online First 5 June 2006].

17. Porensky EK, Dew MA, Karp JF, et al. The burden of late-life generalized anxiety disorder: effects on disability, health-related quality of life, and healthcare utilization. *Am J Geriatr Psychiatry* 2009;17:473-82 [published Online First 30 July 2012].

18. Munk-Jorgensen P, Allgulander C, Dahl AA, et al. Prevalence of generalized anxiety disorder in general practice in Denmark, Finland, Norway, and Sweden. *Psychiatr Serv* 2006;57):1738-44 doi: 10.1176/ps.2006.57.12.1738 [published Online First 28 February 2005].

19. Belanger L, Ladouceur R, Morin CM. Generalized anxiety disorder and health care use. *Can Fam Physician* 2005;51:1362-3.

20. Wittchen HU, Kessler RC, Beesdo K, et al. Generalized anxiety and depression in primary care: prevalence, recognition, and management. *J Clin Psychiatry* 2002;63:24.

21. Day N, Oakes S, Luben R, et al. EPIC-Norfolk: study design and characteristics of the cohort. European Prospective Investigation of Cancer. *Br J Cancer* 1999;80:95-103.

22. Hayat SA, Luben R, Keevil VL, et al. Cohort profile: a prospective cohort study of objective physical and cognitive capability and visual health in an ageing population of men and women in Norfolk (EPIC-Norfolk 3). *Int J Epidemiol* 2014;43:1063-72 doi: 10.1093/ije/dyt086 [published Online First 14 June 2013].

For peer review only

23. Surtees P, Wainwright N, Khaw K, et al. Functional health status, chronic medical conditions and disorders of mood. *Br J Psychiatry* 2003;183:299–303 doi: 10.1192/bjp.183.4.299 [published Online First 30 September 2003].

24. Leng Y, Wainwright NWJ, Cappuccio FP, et al. Daytime napping and the risk of all-cause and cause-specific mortality: a 13-year follow-up of a British population. *Am J Epidemiol* 2014;179:1115-1124 doi: https://doi.org/10.1093/aje/kwu036 [published Online First 30 March 2014].

25. Leng Y, Wainwright NWJ, Cappuccio FP, et al. Self-reported sleep patterns in a British population cohort. *Sleep Med* 2014;15:295-302 doi: 10.1016/j.sleep.2013.10.015.

26. Leng Y, Wainwright NW, Cappuccio FP, et al. Daytime napping and increased risk of incident respiratory diseases: symptom, marker, or risk factor? *Sleep Med* 2016;23:12-15 doi: 10.1016/j.sleep.2016.06.012 [published Online First 12 July 2016].

27. Shohaimi S, Luben R, Wareham N, et al. Residential area deprivation predicts smoking habit independently of individual educational level and occupational social class. A cross sectional study in the Norfolk cohort of the European Prospective Investigation into Cancer (EPIC-Norfolk). *J Epidemiol Community Health* 2003;57:270-276 doi: 10.1136/jech.57.4.270.

28. Myint PK, Surtees PG, Wainwright NW, et al. Physical health-related quality of life predicts stroke in the EPIC-Norfolk. *Neurology* 2007;69:2243-8 doi: 10.1212/01.wnl.0000296010.21252.78.

29. Elias P, Halstead K, Prandy K. CASOC: Computer-assisted standard occupational coding. London, England: HMSO 1993.

For peer review only

30. Surtees PG, Wainwright NWJ, Brayne C. Psychosocial aetiology of chronic disease: a pragmatic approach to the assessment of lifetime affective morbidity in an EPIC component study. *J Epidemiol Community Health* 2000;54:114-122 doi: 10.1136/jech.54.2.114.

31. Remes O, Brayne C, Van der Linde R, et al. A systematic review of reviews on the prevalence of anxiety disorders in adult populations. *Brain Behav* 2016;6:e00497 doi: 10.1002/brb3.497 [published Online First 5 June 2016].

32. Ware JE, Snow K, Kosinski M, et al. New England Medical Center Hospital. Health Institute. SF-36 Health Survey: Manual and Interpretation Guide. The Health Institute; Boston, MA: New England Medical Center 1993.

33. Lakshman R, McConville A, How S, et al. Association between area level socio-economic deprivation and a cluster of behavioural risk factors: Cross sectional, population based study. *J Public Health (Oxf)* 2011;33:234-245. doi: 10.1093/pubmed/fdq072 [published Online First 29 September 2010].

34. Vetter S, Endrass J, Schweizer I, et al. The effects of economic deprivation on psychological well-being among the working population of Switzerland. *BMC Public Health* 2006;6:223. doi: 10.1186/1471-2458-6-223 [published Online First 4 September 2006].

35. Luppa M, Sikorski C, Motzek T, et al. Health service utilization and costs of depressive symptoms in late life – a systematic review. *Curr Pharm Des* 2012;18:5936-57.

36. Ford JD, Trestman RL, Steinberg K, et al. Prospective association of anxiety, depressive, and addictive disorders with high utilization of primary, specialty and emergency medical care. *Soc Sci Med* 2004;58:2145-8 doi: 10.1016/j.socscimed.2003.08.017 [published Online First 25 September 2003].

For peer review only

37. Deacon B, Lickel J, Abramowitz JS. Medical utilization across the anxiety disorders. *J Anxiety Disord* 2008;22:344-50 doi: 10.1016/j.janxdis.2007.03.004 [published Online First 12 March 2007].

38. Klassen BJ, Porcerelli JH, Markova T. The effects of PTSD symptoms on health care resource utilization in a low-income, urban primary care setting. *J Trauma Stress* 2013;26:636-9 doi: 10.1002/jts.21838 [published Online First 12 September 2013].

For peer review only

Figure 1 – Flowchart of EPIC-Norfolk cohort

This is a flowchart showing the number of participants at each study stage: the number approached to participate in the EPIC-Norfolk study, the number enrolled at baseline, and with complete data on all covariates. The EPIC-Norfolk study consists of middle-aged and older British people.

For peer review only

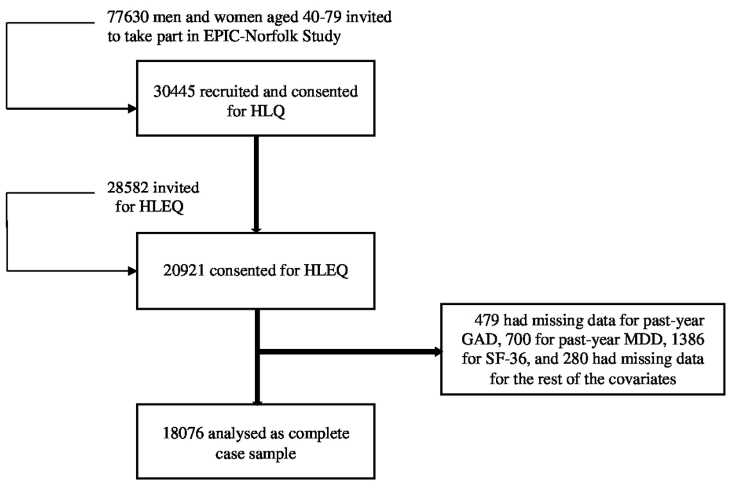

Figure 1: Flowchart of EPIC-Norfolk cohort

215x279mm (200 x 200 DPI)

Please see the article line numbers (column on the right) and the explanations provided.

STROBE Statement—checklist of items that should be included in reports of observational studies

	Item No	Recommendation	Line numbers within the article
**Title and abstract**	1	(*a*) Indicate the study's design with a commonly used term in the title or the abstract	9, 40, 43
		(*b*) Provide in the abstract an informative and balanced summary of what was done and what was found	35-68
**Introduction**			
Background/rationale	2	Explain the scientific background and rationale for the investigation being reported	95-144
Objectives	3	State specific objectives, including any prespecified hypotheses	146-150
**Methods**			
Study design	4	Present key elements of study design early in the paper	157-158
Setting	5	Describe the setting, locations, and relevant dates, including periods of recruitment, exposure, follow-up, and data collection	158-169
Participants	6	(*a*) *Cohort study*—Give the eligibility criteria, and the sources and methods of selection of participants. Describe methods of follow-up<br>*Case-control study*—Give the eligibility criteria, and the sources and methods of case ascertainment and control selection. Give the rationale for the choice of cases and controls<br>*Cross-sectional study*—Give the eligibility criteria, and the sources and methods of selection of participants	158-160, 162-165
		(*b*) *Cohort study*—For matched studies, give matching criteria and number of exposed and unexposed<br>*Case-control study*—For matched studies, give matching criteria and the number of controls per case	
Variables	7	Clearly define all outcomes, exposures, predictors, potential confounders, and effect modifiers. Give diagnostic criteria, if applicable	175-252
Data sources/ measurement	8	For each variable of interest, give sources of data and details of methods of assessment (measurement). Describe comparability of assessment methods if there is	There were two variables of interest in this study: generalized anxiety disorder and hospital admissions

For peer review only

For peer review only - http://bmjopen.bmj.com/site/about/guidelines.xhtml

		more than one group	(175-185, 226-252). The others are potential confounders – in the methods I list them all, indicate how they were assessed and mention that they were collected through the baseline, self-reported postal HLQ questionnaire as well as the HLEQ.
Bias	9	Describe any efforts to address potential sources of bias	278 (complete case analysis)
Study size	10	Explain how the study size was arrived at	276-278
Quantitative variables	11	Explain how quantitative variables were handled in the analyses. If applicable, describe which groupings were chosen and why	In the methods, I mention how the variables were derived based on the raw data provided by participants in the questionnaires.<br>Confounders: 193-212; Generalized anxiety disorder: 180-185; Information on how health service use data was handled in analyses: 260-262
Statistical methods	12	(*a*) Describe all statistical methods, including those used to control for confounding	254-278
		(*b*) Describe any methods used to examine subgroups and interactions	We did not examine any subgroups or interactions.
		(*c*) Explain how missing data were addressed	We indicated that this was a complete-case analysis.
		(*d*) *Cohort study*—If applicable, explain how loss to follow-up was addressed<br>*Case-control study*—If applicable, explain how matching of cases and controls was addressed<br>*Cross-sectional study*—If applicable, describe analytical methods taking account of sampling strategy	Loss to follow-up was not a problem in this study. We were able to track down all participants using various means, unless they expressed that they wished to be removed from the mailing list. We elaborate on this in the manuscript.
		(*e*) Describe any sensitivity analyses	There were no sensitivity analyses.

For peer review only

For peer review only - http://bmjopen.bmj.com/site/about/guidelines.xhtml

**Results**			
Participants	13*	(a) Report numbers of individuals at each stage of study—eg numbers potentially eligible, examined for eligibility, confirmed eligible, included in the study, completing follow-up, and analysed	287-291
		(b) Give reasons for non-participation at each stage	We do not have the reasons for non-participation, because these data were not collected when the study was initiated in 1993.
		(c) Consider use of a flow diagram	Flow diagram included in submission.
Descriptive data	14*	(a) Give characteristics of study participants (eg demographic, clinical, social) and information on exposures and potential confounders	293-294, 296-312, 314-317; we provided characteristics for those with vs. without GAD, because we felt it was important to show the characteristics of the 'exposed' vs. 'non-exposed' group (see also Table 1)
		(b) Indicate number of participants with missing data for each variable of interest	287-289
		(c) *Cohort study*—Summarise follow-up time (eg, average and total amount)	291
Outcome data	15*	*Cohort study*—Report numbers of outcome events or summary measures over time	293
		Case-control study—Report numbers in each exposure category, or summary measures of exposure	
		Cross-sectional study—Report numbers of outcome events or summary measures	
Main results	16	(*a*) Give unadjusted estimates and, if applicable, confounder-adjusted estimates and their precision (eg, 95% confidence interval). Make clear which confounders were adjusted for and why they were included	Table 3 contains unadjusted and progressively adjusted estimates. We also discussed the findings within the text, and provide odds ratios and 95% confidence intervals. We included the confounders based on the literature – we mention this in the paper. As per strobe, we included this information in the methods section; and we omitted repeating this in the results section to reduce redundancy. However, if the editor would like us to repeat this information in the results, we are happy to do so.
		(*b*) Report category boundaries when continuous variables were categorized	The age cut-offs are provided. In regards to the

For peer review only - http://bmjopen.bmj.com/site/about/guidelines.xhtml

			physical component summary of the SF-36, the methods section states that those below and above the median cut-point were compared. The results section (footnotes of the tables) indicates that 3 units of alcohol was the cut point used to differentiate those with ‘high’ versus ‘low’ alcohol intake.
		(*c*) If relevant, consider translating estimates of relative risk into absolute risk for a meaningful time period	
Other analyses	17	Report other analyses done—eg analyses of subgroups and interactions, and sensitivity analyses	Not applicable.
**Discussion**			
Key results	18	Summarise key results with reference to study objectives	375-379, 388-390
Limitations	19	Discuss limitations of the study, taking into account sources of potential bias or imprecision. Discuss both direction and magnitude of any potential bias	400-424
Interpretation	20	Give a cautious overall interpretation of results considering objectives, limitations, multiplicity of analyses, results from similar studies, and other relevant evidence	465-490 and 505-508 (We also have a section comparing our study results to those of others: 426-450 as well as a section on potential mechanisms explaining our findings: 452-463)
Generalisability	21	Discuss the generalisability (external validity) of the study results	423-424
**Other information**			
Funding	22	Give the source of funding and the role of the funders for the present study and, if applicable, for the original study on which the present article is based	518-519

*Give information separately for cases and controls in case-control studies and, if applicable, for exposed and unexposed groups in cohort and cross-sectional studies.

Note: An Explanation and Elaboration article discusses each checklist item and gives methodological background and published examples of transparent reporting. The STROBE checklist is best used in conjunction with this article (freely available on the Web sites of PLoS Medicine at http://www.plosmedicine.org/, Annals of Internal Medicine at http://www.annals.org/, and Epidemiology at http://www.epidem.com/). Information on the STROBE Initiative is available at www.strobe-statement.org.

For peer review only

For peer review only - http://bmjopen.bmj.com/site/about/guidelines.xhtml

BMJ Open

Generalized anxiety disorder and non-psychiatric hospital admissions: findings from a large, population study

Journal:	*BMJ Open*
Manuscript ID	bmjopen-2017-018539.R1
Article Type:	Research
Date Submitted by the Author:	27-Sep-2017
Complete List of Authors:	Remes, Olivia; University of Cambridge, Public Health and Primary Care<br>Wainwright, Nicholas; University of Cambridge, UK, Public Health and Primary Care (retired)<br>Surtees, Paul; University of Cambridge, Department of Public Health and Primary Care<br>LaFortune, Louise; University of Cambridge, Institute of Public Health<br>Khaw, Kay-Tee; University of Cambridge, Department of Public Health and Primary Care<br>Brayne, Carol; University of Cambridge, Institute of Public Health
**Primary Subject Heading**:	Mental health
Secondary Subject Heading:	Health services research, Public health
Keywords:	EPIDEMIOLOGY, MENTAL HEALTH, PUBLIC HEALTH

SCHOLARONE™
Manuscripts

For peer review only

Generalized anxiety disorder and non-psychiatric hospital admissions: findings from a large, population study

Olivia Remes[1], Nick Wainwright[2], Paul Surtees[1], Louise Lafortune[1], Kay-Tee Khaw[3], Carol Brayne[1]

[1]Department of Public Health and Primary Care, University of Cambridge, Forvie Site, Robinson Way, Cambridge, UK CB2 0SR

[2](Retired) Department of Public Health and Primary Care, University of Cambridge, 2 Worts' Causeway, Cambridge, UK CB1 8RN

[3]Department of Public Health and Primary Care, University of Cambridge, 2 Worts' Causeway, Cambridge, UK CB1 8RN

Figures: 1; Tables: 4

Correspondence to: Olivia Remes or260@medschl.cam.ac.uk, telephone: 07776740550

For peer review only

ABSTRACT

OBJECTIVE

Generalized anxiety disorder (GAD) is the most common anxiety disorder in the general population, and has been associated with high economic and human burden. However, it has been neglected in the health services literature. The objective of this study will be to assess whether GAD leads to non-psychiatric hospital admissions using data from the European Prospective Investigation of Cancer-Norfolk. Other aims include determining whether early or late onset forms of the disorder, episode chronicity and frequency, and comorbidity with major depressive disorder (MDD) contribute to non-psychiatric hospital admissions.

DESIGN

Large, population study.

SETTING

UK population-based cohort.

PARTICIPANTS

30,445 people over the age of 40 were recruited through general practice registers in England. Of these, 20,919 completed a structured health and lifestyle questionnaire used to assess GAD. Anxiety was examined in 1996-2000, and health service use was captured between 1999/00 and 2009 through record linkage with large, administrative health databases. 17,939 participants had complete data on covariates.

MAIN OUTCOME MEASURE

Past-year GAD defined according to the Diagnostic and Statistical Manual of Mental Disorders, fourth edition.

RESULTS

In this study, 2.2% (393/17,939) respondents had GAD. Anxiety was not independently associated with non-psychiatric hospital admissions (IRR=1.04, 95% CI: 0.90, 1.20) over nine years. However, those who developed anxiety before 30 years of age seemed to be at increased risk for hospital service use (IRR=1.16, 95% CI: 0.95, 1.41).Those whose anxiety was comorbid with depression showed a statistically significant association with non-psychiatric hospital admissions (IRR=1.23, 95% CI: 1.02, 1.49).

CONCLUSION

People with an earlier age of GAD onset and who had MDD comorbidity were at an increased risk for hospital admissions. Clinicians should consider that it may not be the diagnosis of the disorder at one point in time that is predictive of deleterious health outcomes; rather its long-term course and different forms of the disorder might be of greater importance.

Key words: Anxiety, anxiety disorders, health services

For peer review only

For peer review only - http://bmjopen.bmj.com/site/about/guidelines.xhtml

ARTICLE SUMMARY

Strengths and limitations of this study

- We used a large, population-based sample of middle- and older-aged adults and adjusted for a range of important confounders, such as, sociodemographic factors and medical history.
- We used a structured, self-reported questionnaire to assess presence of past-year GAD, and participants were followed for 9 years.
- We examined health services through record linkage with large, administrative health databases.
- Those who participated in this study were somewhat less deprived and healthier than individuals living in other parts of England; therefore, our results may not generalize to people living in extremely deprived circumstances.

For peer review only

INTRODUCTION

Anxiety disorders[1] are the most common class of psychiatric disorders in the general population. The Global Burden of Disease study[2] estimated that anxiety disorders contribute to 26.8 million disability adjusted life years, and their annual direct cost is $42.3 billion[3]. Generalized anxiety disorder (GAD) is characterized by excessive, pervasive worry, and a number of additional symptoms, such as restlessness and muscle tension. It is a prevalent and disabling condition in adults, and can lead to serious impairment in social and occupational functioning.[4] GAD is associated with poor quality of life, impaired functioning and risk of suicide.[5-8] Across the anxiety disorders, this condition has been found to be the most debilitating.[5, 9] Although there is effective treatment for GAD, only a third of those affected receive any treatment.[8] This is because anxiety disorders are frequently under-recognized and mismanaged by clinicians in primary care, which is often the first point of contact for those with mental health problems.[10]

Although detection of anxiety in clinical settings is poor[11, 12] and the presence of undiagnosed mental health problems can contribute to further emotional distress in patients down the line[12], it could be that disorders such as GAD represent more than just psychological or worry-related symptoms. It may be that anxiety symptoms are masking underlying poor physical health or could be an early warning signal for future health problems that are not yet detectable by standard medical tests. Such problems cannot be simply resolved through psychological therapies or psychotropic medication.

Anxiety has been linked to hypothalamic-pituitary-adrenal (HPA)-axis dysregulation and inflammation, and this can lead to poor health.[9] A recent study of hospitalized patients[13] also showed that people with anxiety disorders had more co-morbid physical conditions, including cardiovascular diseases and their risk factors, compared to people without anxiety disorders. Conversely, anxiety could also represent a response to underlying medical illness, and physical illness can exacerbate anxiety; the possibility of a bidirectional relationship between anxiety and physical health should not be excluded.[14, 15] Compelling evidence from prospective studies, however, has shown that anxiety can

For peer review only

indeed increase the risk of serious chronic conditions, such as cancer[16] and coronary heart disease (CHD)[17].

When investigating the links between mental disorders and health outcomes, early or late-onset forms of anxiety disorders, as well as psychiatric comorbidity should be also considered. A study[18] of over one million Swedish men followed for over 20 years showed that early-onset forms of mental disorders in particular led to increased risk of incident CHD. Anxiety disorders, such as, GAD are also frequently comorbid with major depressive disorder (MDD)[19], and psychiatric comorbidity has been associated with poorer quality of life, worse prognosis, and higher use of health services for mental health problems than pure forms of the disorder.[20, 21, 22] Therefore, identifying clinical aspects, such as, early or late onset forms of the condition, episode chronicity and frequency, and comorbidity with MDD can lead to better clinical management and more accurate prediction of future disability and health service use.[23]

GAD is one of the most common anxiety disorders in the general population[24] and the primary care setting[25], and has been associated with high economic and human burden. However, it has been neglected in the health services literature, with the exception of some studies showing GAD to contribute to higher use of primary care services in primary care samples.[26-29] Clinical samples, however, have the potential for self-selection bias. Whether GAD leads to non-psychiatric hospital admissions is unknown.

The objective of this study will be to assess the association between GAD and non-psychiatric hospital admissions in a longitudinal, population cohort of over 18,000 British individuals followed for 10 years. The aim is also to determine whether early or late onset forms of the disorder, episode frequency and chronicity, and comorbidity with MDD contribute to non-psychiatric hospital admissions.

For peer review only

METHODS

Study population

The study population was drawn from the European Prospective Investigation of Cancer-Norfolk longitudinal, cohort study, described in detail elsewhere.[30, 31] Briefly, a total of 30,445 participants over the age of 40 living in Norwich and the surrounding towns and rural areas were recruited between 1993 and 1997 using general practice registers. At baseline, they completed a health questionnaire capturing sociodemographics and medical history. During follow-up, between 1993 and 1999/2000, participants completed self-reported postal questionnaires provided they: 1) were still alive, 2) did not ask to be removed from the study's mailing list, and 3) had a valid mailing address. Between 1996 and 1999/2000, respondents completed a Health and Life Experiences Questionnaire (HLEQ)[30] used to capture information on psychiatric disorders, other psychosocial factors, and risk behaviours. Record linkage with administrative health databases using a unique identifier was used to determine hospitalization admissions data until 2009.

All participants recruited through general-practice registers and who completed a baseline health questionnaire were eligible to be included in our study; those who completed a psychosocial questionnaire during follow-up were eligible to be included in our analysis.

Assessment of GAD

The HLEQ was used to derive a measure of GAD according to the Diagnostic and Statistical Manual of Mental Disorders, fourth edition. The HLEQ captured the onset and offset timings of episodes of past-year GAD.[32] Past-year GAD consisted of at least one episode that had offset within 12 months of administration of the HLEQ. DSM-IV GAD was diagnosed if participants reported having uncontrollable, excessive worry for six months or longer on most days than not that resulted in disability or impairment. In addition, at least three of the following symptoms needed to have been present: restlessness, irritability, muscle tension, fatigue, trouble concentrating because of worry, mind going blank, trouble falling asleep, trouble staying asleep, and feeling keyed up or on edge.

For peer review only

Assessment of covariates

Potential confounders (based on the literature) included sociodemographics (age, sex, education, marital status, social class, employment), prevalent physical diseases, disability, MDD, and risk behaviours (alcohol use, smoking, physical activity). The final categorization of the variables took cell size into account and was also done in accordance with previous literature.[32, 33-38] Age was first assessed as a categorical variable, and subsequently divided into 10-year bands. Sex was categorized into male vs. female; marital status was categorized into: married, single (or never married), and others (widowed, divorced, separated); educational attainment into high (vocational or formal qualifications at the A- or O-level or degree-level qualifications) vs. low (no formal qualifications). Social class was derived using the Computer-Assisted Standard Occupational Coding[38] and categorized as follows: I (professionals), II (managerial and technical occupations), III non-manual and III manual (skilled workers), IV (partly skilled workers), and V (unskilled manual workers). For men, social class was coded using their own occupation except when they were unemployed or retired in which case their partner‘s social class was used. Unemployed men without partners were unclassified. Social class in women was based on their partner‘s except when the partner‘s social class was unclassified, missing, or they had no partner in which case social class was based on their own occupation. An unemployed woman without a partner was coded as unclassified. The final categorization of social class included manual: skilled manual, partly skilled, and unskilled; and non-manual: professionals, managerial and technical, and skilled non-manual. Employment was divided into yes vs. no.

Behaviour risk factor measures included alcohol intake (units of alcohol/week), smoking status (current, former, non-smoker), and physical activity (inactive, moderately inactive, moderately active, active). Presence of past-year DSM-IV major depressive disorder (MDD) (yes/no) was also assessed.[39]

Individual-level health status was assessed through the construction of a variable capturing major prevalent physical diseases associated with anxiety.[40] This was based on HLQ questions asking participants: “Has the doctor ever told you that you have any of the following?”, followed by a list of options, such as stroke, myocardial infarction, and cancer.

For peer review only

To determine disability levels, we used the physical component summary score (PCS) of the Medical Outcomes Study 36-Item Short Form (SF-36), a widely-used, validated self-assessment tool. A score of 100 represents no disability and 0 represents a high level of disability.[41] PCS scores were dichotomized above and below the median.

All of these individual-level variables were regarded as potential confounders and selected based on the literature and their association with anxiety and health service use.[42, 43]

Hospital service use

All analyses are based on non-psychiatric hospitalizations. Primary care service use was not captured in this study.

Frequency of hospitalization between 1999/00 and 2009 was determined using administrative health databases maintained by the National Health Service. The East Norfolk Primary Health Care trust databases were used, and these are updated on an ongoing basis and provide information on clinical and administrative data from participating facilities, such as, hospitals.

England is under a publicly-funded health care system (the National Health Service), free at the point of delivery; therefore, we expect factors, such as access to health insurance or personal income, to have minimal impact on the care that is obtained by study participants. The databases used in this study are maintained by the National Health Service, which is likely to capture most hospital admissions from the population, as private sector provision is minimal. This means that admissions data in our study can be considered complete for the ascertainment of hospital/health service use, and the likelihood of bias minimal. To access hospital services in the UK, a referral is needed from the primary care practitioner, who acts as a gate-keeper to secondary care.

The East Norfolk Primary Health Care databases were linked to the EPIC-Norfolk cohort using participants' unique National Health Service number, which allows complete record linkage across settings and calendar time.

For peer review only

Vital status for participants was determined through record linkage with the United Kingdom Office of National Statistics. Vital status was available for all participants. This allowed us to exclude those who died before their health service use was ascertained.

Statistical Analysis

First, demographics, social class, medical and psychiatric conditions, and risk behaviours were compared by GAD status - the chi-square test was used to determine whether differences were statistically significant for categorical variables. Second, the mean number of hospital admissions was determined for each characteristic/covariate - the Kruskal Wallis test was used to determine statistical significance for categorical covariates with three or more categories, while the Wilcoxon rank-sum test was used for dichotomous covariates.

Since the number of hospital admissions was skewed and the variance was much larger than the mean, zero-inflated negative binomial regression was used for frequency of hospital utilization (number of hospital admissions). The log-likelihood test showed that this model was superior to Poisson regression. Three models were fitted for hospital admissions with progressive adjustment of covariates: model A adjusted for sociodemographics (age, sex, education, marital status, social class, employment), physical conditions and disability; model B further accounted for past-year MDD (assessed at the same questionnaire point as past-year GAD); and model C further controlled for physical activity, alcohol, and smoking. Multiple imputations for missing data were also carried out on the fully-adjusted model assessing the association between past-year GAD and non-psychiatric hospitalization (our primary objective).

Finally, we determined whether the risk for hospitalization was higher among those with: 1) 3 or more episodes of lifetime GAD (versus those with fewer than 3 episodes or no GAD), 2) episodes that lasted on average 6 months or more (versus those with fewer than 6 months or no GAD), 3) age of onset at 30 years or younger (versus people with age at onset over 30 years or no GAD), and 4) psychiatric comorbidity with MDD (versus no GAD-MDD comorbidity). Two-sided statistical tests for the maximum likelihood zero inflation

For peer review only

parameter estimates were conducted and a p-value of <0.05 was used for statistical significance. Analyses were implemented in SAS, version 9.3.

To arrive at the study size, we went through the following steps: of the 30,445 who completed the baseline HLQ, we retained those participants who completed the HLEQ (20,919), and of these, we kept those people with complete data on all covariates (17,939). (Figure 1)

Patient involvement

There were no patients involved in the development of the research question and outcome measures, the design of the study, or the recruitment to and conduct of the study.

For peer review only

RESULTS

Of the 30,445 people recruited at baseline, 20,919 participants completed the HLEQ; most of the missing observations were from past-year GAD (479), past-year MDD (700), and disability (1386); the rest of the missing observations were generated from the other covariates (Figure 1). Notable findings from the missing data analysis show that people with missing GAD more often had pre-existing health conditions, high disability, MDD, low alcohol consumption, and were without employment (Appendix I).

The final sample included a total of 17,939 participants. Participants were assessed between 1999/00 and 2009 (followed for 9 years) (Figure 1).

In 1996-2000, GAD was present in 393 out of 17,939 (2.2%) people. Table 1 shows the baseline characteristics of participants by GAD status.

For peer review only

Table 1 Percentage and number of people with past-year GAD reported in 1996-2000 according to sociodemographic factors, health status, and behaviour risk factors for the EPIC-Norfolk cohort (n=17,939)

Characteristic	Number with characteristic	Percentage and number with past-year GAD
**Socio-demographics**		
**Age (years)**		
<50	2359	3.4 (79)[a]
50-60	6209	2.9 (179)
60-70	5733	1.6 (94)
70+	3638	1.1 (41)
**Sex**		
Women	9937	2.5 (249)[b]
Men	8002	1.8 (144)
**Education**[‡]		
Low	6106	2.0 (120)[b]
High	11833	2.3 (273)
**Marital status**		
Single	686	3.6 (25)[a]
Married	14538	2.0 (284)
Other[*]	2715	3.1 (84)
**Social class**		
Manual	6836	2.0 (137)
Non-manual	11103	2.3 (256)
**Employment**		
Yes	7712	2.0 (155)
No	10227	2.3 (238)
**Health status**		
**Physical conditions**[+]		
Yes	9166	2.7 (251)[a]
No	8773	1.6 (142)
**Disability level**		
High[¶]	8900	3.0 (266)[a]
Low	9039	1.4 (127)
**Psychiatric conditions**		
**Past-year MDD**		
Yes	934	21.4 (200)[a]
No	17005	1.1 (193)
**Behaviour risk factors**		
**Physical activity**		
Active[¥]	12822	2.1 (272)
Inactive	5117	2.4 (121)
**Smoking status**		
Current smoker	1893	4.7 (89)[a]
Former smoker	7470	1.9 (141)
Never smoker	8576	1.9 (163)
**Alcohol intake**		
High[α]	9241	2.0 (182)[b]

For peer review only

For peer review only - http://bmjopen.bmj.com/site/about/guidelines.xhtml

Low	8698	2.4 (211)

‡ High education: O-level, A-level, degree; low education: refers to no education

* Other: divorced, separated, widowed

+ Physical conditions: respiratory disease (asthma and bronchitis), allergies and hay fever, stroke, heart attack, cancer, diabetes, thyroid conditions, arthritis

¶ Below the median PCS value of 50.6

¥ Moderately inactive, moderately active, active

α 3+ units of alc./week (1 pint beer=2 units, 1 glass wine=1 unit, 1 glass sherry=1 unit, 1 glass spirits=1 unit)

[a] $P < 0.001$

[b] $P < 0.05$

For peer review only

Those with GAD were more likely to be younger, women, inactive, current smokers, low alcohol consumers, of higher educational attainment, single, of non-manual social class, without employment, with physical conditions, high levels of disability, and MDD. Table 2 summarizes the means and standard deviations of the number of hospital admissions by participant characteristics.

For peer review only

Table 2: Non-psychiatric hospital admissions (mean, SD) by participant characteristics in 17,939 British people between 1999/00 and 2009

	Total number with characteristic	Number of admissions
**Characteristic**		**Mean (SD)**
**Past-year GAD**		
Yes	393	4.0 (6.3)[a]
No	17546	3.4 (13.0)
**Socio-demographics**		
**Age (years)**		
<50	2359	1.9 (9.8)[a]
50-60	6209	3.0 (16.5)
60-70	5733	3.8 (11.2)
70+	3638	4.6 (9.6)
**Sex**		
Women	9937	3.1 (14.0)[a]
Men	8002	3.9 (11.3)
**Education[‡]**		
Low	6106	4.1 (17.1)[a]
High	11833	3.1 (10.1)
**Marital status**		
Single	686	3.0 (8.9)[a]
Married	14538	3.3 (10.9)
Other[*]	2715	4.0 (21.0)
**Social class**		
Manual	6836	4.0 (18.3)[a]
Non-manual	11103	3.1 (7.8)
**Employment**		
Yes	7712	2.5 (9.1)[a]
No	10227	4.1 (15.1)
**Health status**		
**Physical conditions[+]**		
Yes	9166	3.9 (10.4)[a]
No	8773	3.0 (15.1)
**Disability level**		
High[¶]	8900	4.4 (16.5)[a]
Low	9039	2.5 (7.8)
**Psychiatric conditions**		
**Past-year MDD**		
Yes	934	4.5 (13.6)[a]
No	17005	3.4 (12.9)
**Behaviour risk factors**		
**Physical activity**		
Active[¥]	12822	3.2 (13.3)[a]
Inactive	5117	4.1 (11.7)
**Smoking status**		
Current smoker	1893	4.6 (26.8)[a]

For peer review only

Former smoker	7470	3.8 (11.4)
Never smoker	8576	2.9 (8.6)
**Alcohol intake**		
High[α]	9241	3.2 (13.3)[a]
Low	8698	3.7 (12.5)

‡ High education: O-level, A-level, degree; low education: refers to no education

* Other: divorced, separated, widowed

+ Physical conditions: respiratory disease (asthma and bronchitis), allergies and hay fever, stroke, heart attack, cancer, diabetes, thyroid conditions, arthritis

¶ Below the median PCS value of 50.6

¥ Moderately inactive, moderately active, active

α 3+ units of alc./week (1 pint beer=2 units, 1 glass wine=1 unit, 1 glass sherry=1 unit, 1 glass spirits=1 unit)

[a] $P < 0.001$

[b] $P < 0.05$

For peer review only

Participants with GAD had a higher frequency of hospitalization compared to those without GAD. Some of the findings show that frequency of hospitalization was markedly higher among older age groups, men, those with low educational attainment, unemployed participants, those with high levels of disability, and with past-year MDD.

Table 3 shows the unadjusted and adjusted incidence rate ratios of hospital admissions by GAD status.

For peer review only

Table 3: Associations between past-year GAD reported in 1996-2000 and non-psychiatric hospital admissions in 1999/00-2009 in 17,939 British people over the age of 40

	IRR and 95% CI			
**Characteristic**	**Crude IRR**	**A[1]**	**B[2]**	**C[3]**
**Past-year GAD**				
Yes	1.18 (1.02, 1.36)	1.25 (1.09, 1.43)	1.10 (0.96, 1.27)	1.04 (0.90, 1.20)
No	1.00	1.00	1.00	1.00
**Socio-demographics**				
**Age**				
Per 10 years	1.36 (1.33, 1.40)	1.19 (1.16, 1.23)	1.20 (1.17, 1.24)	1.21 (1.18, 1.25)
**Sex**				
Women	0.80 (0.76, 0.83)	0.76 (0.73, 0.79)	0.76 (0.72, 0.79)	0.78 (0.74, 0.81)
Men	1.00	1.00	1.00	1.00
**Education‡**				
Low	1.30 (1.24, 1.36)	1.13 (1.08, 1.18)	1.13 (1.08, 1.19)	1.11 (1.06, 1.16)
High	1.00	1.00	1.00	1.00
**Marital status**				
Single	0.88 (0.79, 0.99)	0.85 (0.77, 0.95)	0.85 (0.76, 0.95)	0.84 (0.76, 0.94)
Married	1.00	1.00	1.00	1.00
Other*	1.21 (1.14, 1.28)	1.17 (1.11, 1.24)	1.14 (1.07, 1.21)	1.09 (1.03, 1.16)
**Social class**				
Manual	1.29 (1.23, 1.34)	1.24 (1.19, 1.30)	1.24 (1.19, 1.30)	1.21 (1.16, 1.26)
Non-manual	1.00	1.00	1.00	1.00
**Employment**				
Yes	1.00	1.00	1.00	1.00
No	1.64 (1.57, 1.71)	1.18 (1.12, 1.25)	1.18 (1.12, 1.24)	1.15 (1.09, 1.21)
**Health status**				
**Physical conditions+**				
Yes	1.32 (1.26, 1.37)	1.18 (1.13, 1.23)	1.17 (1.12, 1.22)	1.18 (1.13, 1.23)
No	1.00	1.00	1.00	1.00
**Disability level**				
High¶	1.78 (1.71, 1.86)	1.52 (1.45, 1.59)	1.51 (1.44, 1.57)	1.48 (1.42, 1.55)
Low	1.00	1.00	1.00	1.00
**Psychiatric conditions**				
**Past-year MDD**				
Yes	1.34 (1.22, 1.48)		1.34 (1.22, 1.48)	1.33 (1.21, 1.46)
No	1.00		1.00	1.00
**Lifestyle**				
**Physical activity**				
Active¥	1.00			1.00
Inactive	1.27 (1.21, 1.33)			1.04 (1.00, 1.09)
**Smoking status**				
Current smoker	1.60 (1.49, 1.72)			1.51 (1.41, 1.62)
Former smoker	1.33 (1.27, 1.39)			1.13 (1.08, 1.18)

Never smoker	1.00		1.00
**Alcohol intake**			
High[α]	0.88 (0.85, 0.92)		0.92 (0.88, 0.96)
Low	1.00		1.00

[1] Model A: adjusted for sociodemographics (age, sex, education, marital status, social class, employment), physical conditions, disability
[2] Model B: adjusted for sociodemographics, physical conditions, disability, MDD
[3] Model C: adjusted for sociodemographics, physical conditions, disability, MDD, physical activity, smoking, alcohol

[‡] High education: O-level, A-level, degree; low education: refers to no education
[*] Other: divorced, separated, widowed
[+] Physical conditions: respiratory disease (asthma and bronchitis), allergies and hay fever, stroke, heart attack, cancer, diabetes, thyroid conditions, arthritis
[¶] Below the median PCS value of 50.6
[¥] Moderately inactive, moderately active, active
[α] 3+ units of alc./week (1 pint beer=2 units, 1 glass wine=1 unit, 1 glass sherry=1 unit, 1 glass spirits=1 unit)

For peer review only

After adjustment for sociodemographic variables, physical conditions, and disability, GAD was associated with a 25% higher incidence rate of hospitalization (IRR=1.25, 95% CI: 1.09, 1.43). The incidence rate ratio was somewhat attenuated and became statistically non-significant after further adjustment for MDD (IRR=1.10, 95% CI 0.96, 1.27). The effect estimate approached the null after additional adjustment for behaviour risk factors (IRR=1.04, 95% CI: 0.90, 1.20). Finally, we did multiple imputations for missing data (Appendix II). The effect estimate remained the same when we imputed all covariates except for GAD (IRR: 1.04, 95% CI: 1.02, 1.06). The effect estimate remained virtually unchanged when we imputed all covariates including GAD (IRR: 1.05, 95% CI: 1.03, 1.08).

Next, we assessed whether risk for hospital admissions varied by frequency of GAD lifetime episodes, anxiety episode chronicity, GAD age of onset, and whether the hospitalization risk was higher in those with psychiatric comorbidity (with MDD) (table 4). Results are based on fully-adjusted models.

For peer review only

Table 4: Associations between different forms of GAD reported in 1996-2000 and non-psychiatric hospital admissions in 1999/00-2009 in 17,939 British people ages 40+

Characteristic	IRR and 95% CI			
**GAD type**				
**Frequent GAD**				
Yes[a]	1.07 (0.91, 1.27)[e]			
No	1.00			
**Chronic GAD**				
Yes[b]		1.07 (0.85, 1.35)[e]		
No		1.00		
**Early age GAD onset**				
Yes[c]			1.16 (0.95, 1.41)[e]	
No			1.00	
**Comorbid GAD**				1.23 (1.02, 1.49)[e]
Yes[d]				1.00
No				
**Socio-demographics**				
**Age**				
Per 10 years	1.22 (1.18, 1.25)	1.21 (1.18, 1.25)	1.22 (1.18, 1.25)	1.21 (1.17, 1.25)
**Sex**				
Women	0.78 (0.74, 0.81)	0.78 (0.74, 0.81)	0.78 (0.74, 0.81)	0.78 (0.75, 0.82)
Men	1.00	1.00	1.00	1.00
**Education**[‡]				
Low	1.10 (1.06, 1.16)	1.11 (1.06, 1.16)	1.10 (1.06, 1.16)	1.12 (1.07, 1.18)
High	1.00	1.00	1.00	1.00
**Marital status**				
Single	0.84 (0.76, 0.94)	0.84 (0.76, 0.94)	0.84 (0.76, 0.94)	0.83 (0.74, 0.93)
Married	1.00	1.00	1.00	1.00
Other[*]	1.10 (1.03, 1.16)	1.09 (1.03, 1.16)	1.09 (1.03, 1.16)	1.03 (0.97, 1.10)
**Social class**				
Manual	1.21 (1.16, 1.26)	1.21 (1.16, 1.26)	1.21 (1.16, 1.26)	1.21 (1.16, 1.27)
Non-manual	1.00	1.00	1.00	1.00
**Employment**				
Yes	1.00	1.00	1.00	1.00
No	1.15 (1.09, 1.21)	1.15 (1.09, 1.21)	1.15 (1.09, 1.21)	1.17 (1.11, 1.24)
**Health status**				
**Physical conditions**[+]				
Yes	1.17 (1.13, 1.23)	1.17 (1.13, 1.23)	1.17 (1.13, 1.23)	1.17 (1.12, 1.22)
No	1.00	1.00	1.00	1.00
**Disability level**				
High[¶]	1.48 (1.42, 1.55)	1.48 (1.42, 1.55)	1.48 (1.42, 1.55)	1.48 (1.42, 1.55)
Low	1.00	1.00	1.00	1.00
**Psychiatric conditions**				

For peer review only

For peer review only - http://bmjopen.bmj.com/site/about/guidelines.xhtml

**Past-year MDD**				
Yes	1.32 (1.20, 1.45)	1.33 (1.22, 1.46)	1.33 (1.21, 1.46)	--
No	1.00	1.00	1.00	
**Lifestyle**				
**Physical activity**				
Active[¥]	1.00	1.00	1.00	1.02 (0.97, 1.07)
Inactive	1.04 (1.00, 1.09)	1.04 (1.00, 1.09)	1.04 (1.00, 1.09)	1.00
**Smoking status**				
Current smoker	1.51 (1.41, 1.62)	1.51 (1.41, 1.62)	1.51 (1.41, 1.62)	1.56 (1.45, 1.68)
Former smoker	1.13 (1.08, 1.18)	1.13 (1.08, 1.18)	1.13 (1.08, 1.18)	1.14 (1.09, 1.19)
Never smoker	1.00	1.00	1.00	1.00
**Alcohol intake**				
High[α]	0.92 (0.88, 0.96)	0.92 (0.88, 0.96)	0.92 (0.88, 0.96)	0.93 (0.89, 0.98)
Low	1.00	1.00	1.00	1.00

[a] 3+ episodes of lifetime GAD
[b] GAD episodes lasted at least 6 months
[c] GAD developed before 30 years of age
[d] GAD-MDD comorbidity
[e] Adjusted for sociodemographics, physical conditions, disability, MDD, physical activity, smoking, alcohol

‡ High education: O-level, A-level, degree; low education: refers to no education
* Other: divorced, separated, widowed
+ Physical conditions: respiratory disease (asthma and bronchitis), allergies and hay fever, stroke, heart attack, cancer, diabetes, thyroid conditions, arthritis
¶ Below the median PCS value of 50.6
¥ Moderately inactive, moderately active, active
α 3+ units of alc./week (1 pint beer=2 units, 1 glass wine=1 unit, 1 glass sherry=1 unit, 1 glass spirits=1 unit)

For peer review only

People with more than 3 lifetime episodes had a somewhat higher risk of hospitalization (IRR=1.07, 95% CI: 0.91, 1.27). Those whose episodes lasted, on average, 6 months or longer also had a slight increased risk for admissions compared to those with shorter episodes (IRR=1.07, 95% CI: 0.85, 1.35). People who developed GAD before 30 years of age were 16% more likely to be admitted to the hospital than those who developed it later in life (IRR=1.16, 95% CI: 0.95, 1.41). Finally, we determined whether GAD comorbid with MDD is associated with non-psychiatric hospital admissions. Results showed that people with GAD-MDD comorbidity had a 23% higher chance of being admitted to hospital than people without comorbidity – this association was statistically significant (IRR: 1.23 ,95% CI: 1.02, 1.49).

For peer review only

DISCUSSION

This is the first study to assess the association between GAD and hospital service use in a population-based cohort. This longitudinal study showed that having an episode of GAD in the past year was not independently associated with hospital admissions during the subsequent ten years. However, those who developed GAD at an early age (before 30) and, in particular, those with MDD comorbidity were at an increased risk of being admitted to hospital than those with a later onset and without MDD comorbidity, respectively. The association between GAD-MDD comorbidity and non-psychiatric hospital admissions was statistically significant.

People with past-year GAD were more likely to have medical conditions; nonetheless, including these covariates in the model left the association between past-year GAD and hospital admissions statistically significant. It was only when MDD was introduced in the model as a potential confounder that any remaining association with hospital service utilization was explained away.

Strengths and limitations

There are several strengths associated with our study. We had a large, population-based sample of middle- and older-aged adults and adequately adjusted for a range of possible confounders. We used a structured questionnaire to assess past-year GAD according to DSM-IV criteria, used large administrative health databases to examine hospital service use (avoiding the self-reporting bias found in questionnaire studies), and participants were followed for a long time. We had a large list of self-reported physician diagnoses of chronic diseases that we used to ascertain medical histories. Despite this, the residual effect of diseases not captured by our study, but that are associated with GAD may be present. Past illness may have been underreported, which may have introduced measurement error and further attenuated effect estimates towards the null. A negligible proportion of participants may have obtained care at private facilities, which might have led to non-differential misclassification. The databases used in this study also did not capture admissions to

For peer review only

hospitals outside the UK. However, migration in the EPIC-Norfolk cohort is minimal and does not present a problem.

We may have overadjusted our models with the inclusion of self-evaluated impairment, as this may be part of the expression of psychiatric illness. This can lead to attenuation of effect estimates. If participants chose not to answer certain questions in the HLEQ, this contributed to missing data; however, to avoid biasing the findings, we retained participants for whom we had complete data on all covariates. Non-participation in our study also may have led to non-differential misclassification and further attenuation of effect estimates.

Another limitation is that we did not have data on primary care service use. Merging population cohorts, such as ours, with primary care service administrative databases and hospitalization databases would have provided a more complete picture of the burden of GAD on the health care system.

This study was conducted on people ages 40 years and older and may not be generalizable to younger age groups. We suspect that the strength of the association between GAD-MDD comorbidity and non-psychiatric hospital admissions is weaker for younger populations who are typically healthier than older people. Although young people have a high burden of mental health problems[40, 44], they (especially adolescents) are less likely to have non-psychiatric hospitalizations than older people[45]. It could take many years until the effects of anxiety comorbid with depression accumulate and manifest as poor physical health, thus translating into higher use of non-psychiatric hospital services. As such, we would expect the strength of the association between GAD-MDD comorbidity and hospitalizations to be weaker in young people, however, future studies should investigate this.

Participants were required to complete detailed dietary and lifestyle questionnaires and undergo periodic health assessments. Because those who participated in EPIC-Norfolk were more affluent and healthier than individuals living in other parts of England, our results may not generalize to people living in extremely deprived areas.

For peer review only

Finally, there was missing data in this study. When we conducted multiple imputations for missing data, the effect estimate of our main analysis remained unchanged.

Comparison with other studies

Most of the studies assessing the link between psychiatric disorders and non-psychiatric health service utilization have focused on depression and, to a lesser extent, panic disorder and PTSD, while other anxiety disorders have been significantly underresearched. Most of the studies on depression as a stand alone measure have shown an association with health service use in both clinical and community samples.[46] There are substantially fewer studies on anxiety, and a number of these have shown positive associations with health service use. A US study[47] that recruited patients from an outpatient clinic showed that anxiety disorders were linked to higher utilization of primary care services compared to depressive or addictive disorders. Patients, however, were recruited from an outpatient clinic located in a predominantly rural area, which might have affected generalizability. Another study showed anxiety disorders to be associated with a higher number of consultations in general medical, emergency and specialty settings, such as cardiology and dermatology.[48] In this study, people were sampled from an anxiety clinic, thereby leading to possible selection bias. Other studies showed PTSD and GAD to be associated with health care use, however, this research was based on highly-select samples that have limited generalizability.[26, 28, 49, 50] In contrast to the literature, a major strength of our study was that it was population-based. There is also a lack of research assessing whether the severity of anxiety or different forms of the disorder contribute to even higher health service use rates (early onset forms and comorbid cases are typically the most severe, hardest to treat and with the poorest prognosis [9]).

Mechanisms

A more severe course of GAD can lead to higher rates of health services because of unhealthy behaviours, such as smoking and alcohol (which we controlled for in our analyses). It could also be that a more severe form of anxiety, such as GAD-MDD comorbidity is associated with poorer underlying health, which then leads to higher health

For peer review only

service use rates. Although we controlled for several chronic diseases, we might have missed some conditions that are associated with GAD-MDD comorbidity and hospitalizations. A third explanation for higher health service use in those with comorbid anxiety and depression could relate to inflammatory pathways. If clinically apparent signs of disease have not yet developed in those with psychiatric comorbidity or are at an early, undetectable stage, it will not be possible to measure these factors and adjust for them in analyses.

Implications

GAD is a debilitating and impairing condition.[9] The evidence base on its association with health services is small and confined to clinical settings with the potential for self-selection bias. Our study overcomes many limitations of previous studies, and clarifies that GAD measured at a single point in time (ex. in the past year) is not associated with health service use. Instead, it shows that cases with early age of GAD onset and especially those that are comorbid with depression can lead to increased use of hospital services, after controlling for a range of important confounders. In this study, GAD-MDD comorbidity was associated with a statistically significantly increased risk of hospital admissions.

Population-based research on anxiety is lacking, and thus far, no studies have assessed the association between GAD and non-psychiatric hospitalization. Clinicians should consider that it is not just the diagnosis of the disorder at one point in time that is predictive of deleterious health outcomes; its long-term course and different forms of the disorder may also be important. GAD has a waxing and waning course throughout a patient's life, and many of those affected experience relapse after psychiatric treatment or develop psychiatric comorbidities.

Our findings are important for clinicians and policy-makers. Large numbers of people are affected by anxiety-depression comorbidity and a number of individuals develop anxiety early in life [9]. As such, clinicians should consider more widespread screening for mental health problems and if appropriate, the examination of any underlying health conditions that may require treatment in order to prevent future hospital admissions. Policy-makers

For peer review only

should also consider rolling out more widespread anxiety prevention and screening programmes.

Future research, however needs to examine the reasons for the increased non-psychiatric hospital service use in those with GAD-MDD comorbidity (this can provide additional insight into clinical recommendations). To provide a better understanding of the links between mental and physical health, the bidirectional links between anxiety and physical health problems should also be examined. Finally, future research should merge a population-based cohort with primary and secondary care administrative health databases to provide a more complete picture of the burden of different forms of anxiety on the health care system.

Finally, while our association between early age of GAD onset and hospital admissions did not reach statistical significance, the effect estimate does suggest that people who develop anxiety early in life have a 16% higher chance of being admitted to hospital than those with late-onset cases. Future research should replicate our study with a larger number of anxiety cases by combining common anxiety disorders into one group.[22]

Conclusion

People with an earlier age of onset for GAD and especially those with comorbid MDD had a higher risk for hospital admissions over 9 years between 1996-1999/00 and 2009 in the European Prospective Investigation of Cancer in Norfolk study.

For peer review only

Acknowledgements: OR received a PhD studentship from the National Institute for Health Research.

Competing interest: All authors have completed the ICMJE uniform disclosure form at www.icmje.org/coi_disclosure.pdf and declare: no support from any organisation for the submitted work; no financial relationships with any organizations that might have an interest in the submitted work in the previous three years; no other relationships or activities that could appear to have influenced the submitted work.

Funding: This work was supported by the Medical Research Council UK (grant number SP2024-0201 and SP2024-0204) and Cancer Research UK (grant number G9502233).

Author contributions: OR (corresponding author) had the idea for and conducted the analysis, and wrote the article. CB critically reviewed drafts of the manuscript, KK edited versions of the paper; PS and NW provided feedback into the analysis. OR, CB, KK, LL, PS, and NW contributed to the interpretation of data for the work, agreed to be accountable for all aspects of the work, gave final approval of the version to be published, and made substantial contributions to the analysis and interpretation of data. OR, CB, KK, LL, PS, and NW have seen and approved the final version. OR, CB, KK, LL, PS, and NW had full access to all the data in the study and take responsibility for the integrity of the data and the accuracy of the data analysis. OR acts as guarantor of the study.

Transparency declaration: OR affirms that the manuscript is an honest, accurate, and transparent account of the study being reported; that no important aspects of the study have been omitted; and that any discrepancies from the study as planned have been explained.

Role of study sponsors and statement of independence: The funding sources had no role in the design and conduct of the study; collection, management, analysis, and interpretation of the data; and preparation, review, or approval of the manuscript.

For peer review only

Ethical approval: The study has ethics committee approval from Norfolk Ethics Committee (Rec Ref: 98CN01) and all participants gave informed consent.

Data sharing: No additional data available. Original dataset requests should be sent to the corresponding author. Please contact O Remes at or260@medschl.cam.ac.uk for questions about the statistical code.

Copyright: "The Corresponding Author has the right to grant on behalf of all authors and does grant on behalf of all authors, a worldwide licence to the Publishers and its licensees in perpetuity, in all forms, formats and media (whether known now or created in the future), to i) publish, reproduce, distribute, display and store the Contribution, ii) translate the Contribution into other languages, create adaptations, reprints, include within collections and create summaries, extracts and/or, abstracts of the Contribution, iii) create any other derivative work(s) based on the Contribution, iv) to exploit all subsidiary rights in the Contribution, v) the inclusion of electronic links from the Contribution to third party material where-ever it may be located; and, vi) licence any third party to do any or all of the above."

For peer review only

REFERENCES

1. American Psychiatric Association. (1994). *Diagnostic and Statistical Manual of Mental Disorders* (4th edn). Washington, DC: APA.
2. Whiteford HA, Degenhardt, Rehm J, et al. Global burden of disease attributable to mental and substance use disorders: findings from the global burden of disease study 2010. *Lancet* 2013;13:1575-86 doi: http://dx.doi.org/10.1016/S0140-6736(13)61611-6.
3. Greenberg PE, Sisitsky T, Kessler RC, et al. The economic burden of anxiety disorders in the 1990s. *J Clin Psychiatry* 1999;60:427-35.
4. Kroenke K, Spitzer RL, Williams JB, et al. Anxiety disorders in primary care: prevalence, impairment, comorbidity, and detection. *Ann Intern Med* 2007;146:317-25 doi: 10.7326/0003-4819-146-5-200703060-00004.
5. Comer JS, Blanco C, Hasin DS, et al. Health-related quality of life across the anxiety disorders. *J Clin Psychiatry* 2011;72:43–50 doi: 10.4088/JCP.09m05094blu [published Online First 24 August 2010].
6. Tyrer P, Baldwin D. Generalised anxiety disorder. *Lancet* 2006;16**:**2156–66 doi: http://dx.doi.org/10.1016/S0140-6736(06)69865-6.
7. Hoge E, Ivkovic A, Fricchione G. Generalized anxiety disorder: diagnosis and treatment. *BMJ* 2012;345:e7500 doi: https://doi.org/10.1136/bmj.e7500.
8. Revicki DA, Travers K, Wyrwich KW. Humanistic and economic burden of generalized anxiety disorder in North America and Europe. *J Affect Disord* 2012;140:103–12 doi: 10.1016/j.jad.2011.11.014 [published Online First 9 December 2011].
9. Simpson HB, Neria Y, Lewis-Fernandez R, et al. Anxiety disorders. Theory, research and clinical perspectives. New York, US: Cambridge University Press 2010.
10. Combs H, Markman J. Anxiety disorders in primary care. *Med Clin North Am* 2014;98:1007-23 doi: 10.1016/j.mcna.2014.06.003 [published Online First 11 July 2014].
11. Buszewicz MJ, Chew-Graham C. Improving the detection and management of anxiety disorders in primary care. *Br J Gen Pract* 2011;61:489-490 doi: 10.3399/bjgp11X588259.

For peer review only

12. Wittchen H-U, Muhlig S, Beesdo K. Mental disorders in primary care. *Dialogues Clin Neurosci* 2003;5:115-128.

13. Schoepf D, Heun R. Anxiety disorders and physical comorbidity: increased prevalence but reduced relevance of specific risk factors for hospital-based mortality during a 12.5-year observation period in general hospital admissions. *Eur Arch Psychiatry Clin Neurosci* 2015;265:387-98 doi: 10.1007/s00406-014-0566-9 [published Online First 4 December 2014].

14. Player MS, Peterson LE. Anxiety disorders, hypertension, and cardiovascular risk: a review. *Int J Psychiatry Med* 2011;41:365-377 doi: https://doi.org/10.2190/PM.41.4.f [published Online First 26 October 2011].

15. de Waal MW, Arnold IA, Eekhof JA, van Hemert AM. Somatoform disorders in general practice: prevalence, functional impairment and comorbidity with anxiety and depressive disorders. *Br J Psychiatry* 2004;184:470-6 doi: 10.1192/bjp.184.6.470 [published Online First 1 June 2004].

16. Shen CC, Hu YW, Hu LY, et al. The risk of cancer in patients with generalized anxiety disorder: a nationwide population-based study. *PloS One* 2013;e57399 doi: 10.1371/journal.pone.0057399 [published Online First 27 February 2013].

17. Janszky I, Ahnve S, Lundberg I, Hemmingsson T. Early-onset depression, anxiety, and risk of subsequent coronary heart disease: 37-year follow-up of 49,321 young Swedish men. *J Am Coll Cardiol* 2010;56:31-37. doi: https://doi.org/10.1016/j.jacc.2010.03.033 [published Online First 22 June 2010].

18. Gale CR, Batty GD, Osborn DP, et al. Mental disorders across the adult life course and future coronary heart disease: evidence for general susceptibility. *Circulation* 2014;129:186-93 doi: 10.1161/CIRCULATIONAHA [published Online First 4 November 2013].

19. Carter RM, Wittchen HU, Pfister H, Kessler RC. One-year prevalence of subthreshold and threshold DSM-IV generalized anxiety disorder in a nationally representative sample. *Depress Anxiety* 2001;13:78-88.

20. Lamers F, van Oppen P, Comijs HC, et al. Comorbidity patterns of anxiety and depressive disorders in a large cohort study: the Netherlands Study of Depression and Anxiety (NESDA). *J Clin Psychiatry* 2011;72:341-8 doi: 10.4088/JCP.10m06176blu [published Online First 25 January 2011].

For peer review only

21. Johansson R, Carlbring P, Heedman A, Paxling B, Andersson G. Depression, anxiety and their comorbidity in the Swedish general population: point prevalence and the effect on health-related quality of life. *Peer J* 2013;1:e98 doi: 10.7717/peerj.98 [published Online First 9 July 2013].

22. Hamalainen J, Isometsa E, Sihvo S, Pirkola S, Kiviruusu O. Use of health services for major depressive and anxiety disorders in Finland. *Depress Anxiety* 2008;25:27-37 doi: 10.1002/da.20256 [published Online First 19 January 2007].

23. Maj M. 'Psychiatric comorbidity': an artefact of current diagnostic systems. *Br J Psychiatry* 2005;186:182-84 doi: 10.1192/bjp.186.3.182 [published Online First 28 February 2005].

24. Stein MB. Public health perspectives on generalized anxiety disorder. *J Clin Psychiatry* 2004;65:3-7.

25. Nutt D, Argyropoulos S, Hood S, et al. Generalized anxiety disorder: a comorbid disease. *Eur Neuropsychopharmacol* 2006;16:S109-18 doi: 10.1016/j.euroneuro.2006.04.003 [published Online First 5 June 2006].

26. Porensky EK, Dew MA, Karp JF, et al. The burden of late-life generalized anxiety disorder: effects on disability, health-related quality of life, and healthcare utilization. *Am J Geriatr Psychiatry* 2009;17:473-82 [published Online First 30 July 2012].

27. Munk-Jorgensen P, Allgulander C, Dahl AA, et al. Prevalence of generalized anxiety disorder in general practice in Denmark, Finland, Norway, and Sweden. *Psychiatr Serv* 2006;57):1738-44 doi: 10.1176/ps.2006.57.12.1738 [published Online First 28 February 2005].

28. Belanger L, Ladouceur R, Morin CM. Generalized anxiety disorder and health care use. *Can Fam Physician* 2005;51:1362-3.

29. Wittchen HU, Kessler RC, Beesdo K, et al. Generalized anxiety and depression in primary care: prevalence, recognition, and management. *J Clin Psychiatry* 2002;63:24.

30. Day N, Oakes S, Luben R, et al. EPIC-Norfolk: study design and characteristics of the cohort. European Prospective Investigation of Cancer. *Br J Cancer* 1999;80:95-103.

31. Hayat SA, Luben R, Keevil VL, et al. Cohort profile: a prospective cohort study of objective physical and cognitive capability and visual health in an ageing population

For peer review only

of men and women in Norfolk (EPIC-Norfolk 3). *Int J Epidemiol* 2014;43:1063-72 doi: 10.1093/ije/dyt086 [published Online First 14 June 2013].

32. Surtees P, Wainwright N, Khaw K, et al. Functional health status, chronic medical conditions and disorders of mood. *Br J Psychiatry* 2003;183**:**299–303 doi: 10.1192/bjp.183.4.299 [published Online First 30 September 2003].
33. Leng Y, Wainwright NWJ, Cappuccio FP, et al. Daytime napping and the risk of all-cause and cause-specific mortality: a 13-year follow-up of a British population. *Am J Epidemiol* 2014;179:1115-1124 doi: https://doi.org/10.1093/aje/kwu036 [published Online First 30 March 2014].
34. Leng Y, Wainwright NWJ, Cappuccio FP, et al. Self-reported sleep patterns in a British population cohort. *Sleep Med* 2014;15:295-302 doi: 10.1016/j.sleep.2013.10.015.
35. Leng Y, Wainwright NW, Cappuccio FP, et al. Daytime napping and increased risk of incident respiratory diseases: symptom, marker, or risk factor? *Sleep Med* 2016;23:12-15 doi: 10.1016/j.sleep.2016.06.012 [published Online First 12 July 2016].
36. Shohaimi S, Luben R, Wareham N, et al. Residential area deprivation predicts smoking habit independently of individual educational level and occupational social class. A cross sectional study in the Norfolk cohort of the European Prospective Investigation into Cancer (EPIC-Norfolk). *J Epidemiol Community Health* 2003;57:270-276 doi: 10.1136/jech.57.4.270.
37. Myint PK, Surtees PG, Wainwright NW, et al. Physical health-related quality of life predicts stroke in the EPIC-Norfolk. *Neurology* 2007;69:2243-8 doi: 10.1212/01.wnl.0000296010.21252.78.
38. Elias P, Halstead K, Prandy K. CASOC: Computer-assisted standard occupational coding. London, England: HMSO 1993.
39. Surtees PG, Wainwright NWJ, Brayne C. Psychosocial aetiology of chronic disease: a pragmatic approach to the assessment of lifetime affective morbidity in an EPIC component study. *J Epidemiol Community Health* 2000;54:114-122 doi: 10.1136/jech.54.2.114.
40. Remes O, Brayne C, Van der Linde R, et al. A systematic review of reviews on the prevalence of anxiety disorders in adult populations. *Brain Behav* 2016;6:e00497 doi: 10.1002/brb3.497 [published Online First 5 June 2016].

For peer review only

41. Ware JE, Snow K, Kosinski M, et al. New England Medical Center Hospital. Health Institute. SF-36 Health Survey: Manual and Interpretation Guide. The Health Institute; Boston, MA: New England Medical Center 1993.

42. Lakshman R, McConville A, How S, et al. Association between area level socio-economic deprivation and a cluster of behavioural risk factors: Cross sectional, population based study. *J Public Health (Oxf)* 2011;33:234-245. doi: 10.1093/pubmed/fdq072 [published Online First 29 September 2010].

43. Vetter S, Endrass J, Schweizer I, et al. The effects of economic deprivation on psychological well-being among the working population of Switzerland. *BMC Public Health* 2006;6:223. doi: 10.1186/1471-2458-6-223 [published Online First 4 September 2006].

44. NHS Digital. Adult Psychiatric Morbidity survey. http://content.digital.nhs.uk/catalogue/PUB21748 (accessed 29 September 2016).

45. NHS. Hospital Episode Statistics. http://content.digital.nhs.uk/hes (accessed 10 September 2017)

46. Luppa M, Sikorski C, Motzek T, et al. Health service utilization and costs of depressive symptoms in late life – a systematic review. *Curr Pharm Des* 2012;18:5936-57.

47. Ford JD, Trestman RL, Steinberg K, et al. Prospective association of anxiety, depressive, and addictive disorders with high utilization of primary, specialty and emergency medical care. *Soc Sci Med* 2004;58:2145-8 doi: 10.1016/j.socscimed.2003.08.017 [published Online First 25 September 2003].

48. Deacon B, Lickel J, Abramowitz JS. Medical utilization across the anxiety disorders. *J Anxiety Disord* 2008;22:344-50 doi: 10.1016/j.janxdis.2007.03.004 [published Online First 12 March 2007].

49. Klassen BJ, Porcerelli JH, Markova T. The effects of PTSD symptoms on health care resource utilization in a low-income, urban primary care setting. *J Trauma Stress* 2013;26:636-9 doi: 10.1002/jts.21838 [published Online First 12 September 2013].

50. Lehavot K, Der-Martirosian C, Simpson TL, et al. The role of military social support in understanding the relationship between PTSD, physical health, and healthcare utilization in women veterans. *J Trauma Stress*. 2013;26:772–5 doi: 10.1002/jts.21859 [published Online First 6 November 2013].

For peer review only

Figure 1 – Flowchart of EPIC-Norfolk cohort

This is a flowchart showing the number of participants at each study stage: the number approached to participate in the EPIC-Norfolk study, the number enrolled at baseline, and with complete data on all covariates. The EPIC-Norfolk study consists of middle-aged and older British people.

For peer review only

Figure 1 - Flowchart of EPIC-Norfolk cohort

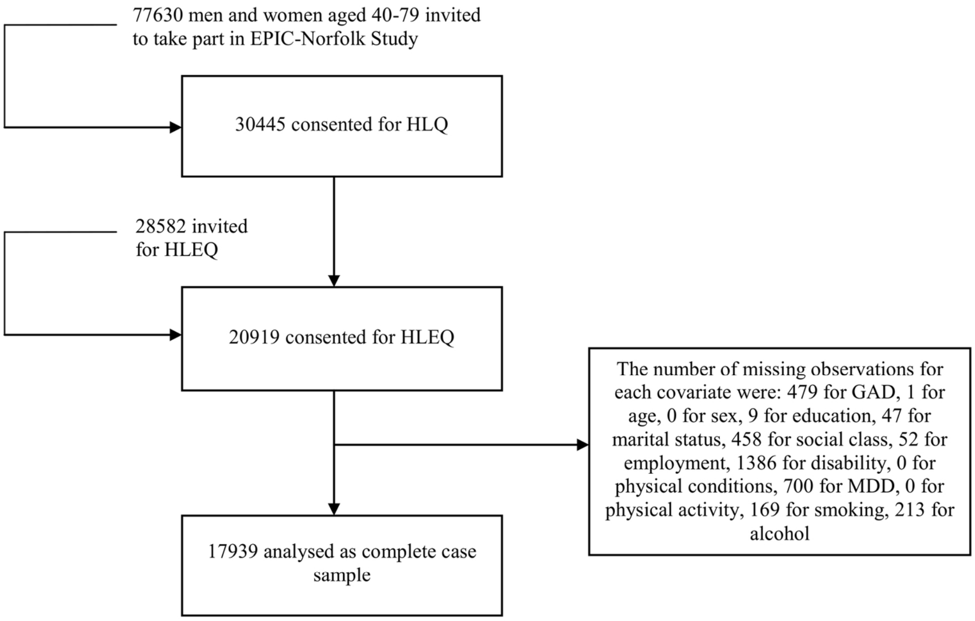

Note: Some participants had missing observations on more than one covariate.

Flowchart of the European Prospective Investigation of Cancer (EPIC)-Norfolk cohort. This is a flowchart showing the number of participants at each study stage: the number approached to participate in the EPIC-Norfolk study, the number enrolled at baseline, and with complete data on all covariates. The EPIC-Norfolk study consists of middle-aged and older British people.

153x135mm (300 x 300 DPI)

Appendix I: Table 1 Percentage and number of people with missing past-year GAD reported in 1996-2000 according to sociodemographic factors, health status, and behaviour risk factors for the EPIC-Norfolk cohort

Characteristic	Total number with characteristic	Percentage and no. with missing past-year GAD
**Socio-demographics**		
**Age (years)**		
<50	2385	1.1 (26)
50-60	6279	1.1 (70)
60-70	5787	0.9 (54)
70+	3685	1.3 (47)
**Sex**		
Women	10055	1.2 (118)
Men	8081	1.0 (79)
**Education**[‡]		
Low	6178	1.2 (72)
High	11958	1.1 (125)
**Marital status**		
Single	695	1.3 (9)
Married	14687	1.0 (149)
Other[*]	2754	1.4 (39)
**Social class**		
Manual	6918	1.2 (82)
Non-manual	11218	1.0 (115)
**Employment**		
Yes	7775	0.8 (63)[b]
No	10361	1.3 (134)
**Health status**		
**Physical conditions**[+]		
Yes	9285	1.3 (119)[b]
No	8851	0.9 (78)
**Disability level**		
High[¶]	9030	1.4 (130)[a]
Low	9106	0.7 (67)
**Psychiatric conditions**		
**Past-year MDD**		
Yes	983	5.0 (49)[a]
No	17153	0.9 (148)
**Behaviour risk factors**		
**Physical activity**		
Active[¥]	12963	1.1 (141)
Inactive	5173	1.1 (56)
**Smoking status**		
Current smoker	1922	1.5 (29)
Former smoker	7543	1.0 (73)

For peer review only

Never smoker	8671	1.1 (95)
**Alcohol intake**		
High[α]	9327	0.9 (86)[b]
Low	8809	1.3 (111)

‡ High education: O-level, A-level, degree; low education: refers to no education
* Other: divorced, separated, widowed
+ Physical conditions: respiratory disease (asthma and bronchitis), allergies and hay fever, stroke, heart attack, cancer, diabetes, thyroid conditions, arthritis
¶ Below the median PCS value of 50.6
¥ Moderately inactive, moderately active, active
α 3+ units of alc./week (1 pint beer=2 units, 1 glass wine=1 unit, 1 glass sherry=1 unit, 1 glass spirits=1 unit)

[a] $P < 0.001$
[b] $P < 0.05$

For peer review only

Appendix 2 – Multiple imputations for missing data

1. Main analysis: association between past-year GAD and non-psychiatric hospital admissions

First, we imputed missing data for all covariates except GAD, our main exposure. Based on the literature, we identified potential auxiliary variables, and we retained those that were correlated with the variables in our model and were good predictors of the missing status (based on statistical tests). Our imputation model included all variables from the analysis model and the auxiliary variables.

To retain as much information as possible, we conducted the imputations on non-transformed data-the original variables in our dataset. We imputed data using the fully conditional specification, and specified a linear regression model for continuous data that were normally distributed; predictive mean matching for continuous data that were not normally distributed; and logistic regression for categorical variables. Variable estimates were subsequently averaged from 100 imputed datasets using Rubin's rules (we transformed the data before running the analytic model of interest within each of the imputed datasets).[1]

We checked whether the imputations were acceptable by comparing 1) the means, standard deviations, and plots of recorded and imputed values for continuous variables, and 2) the frequencies and percentages of recorded and imputed values for each level of categorical variables.

Analyses were done using SAS 9·3 and p-values less than 0·05 were considered statistically significant.

Findings from this set of multiple imputations for missing data analysis provided the following effect estimate relating past-year GAD and non-psychiatric hospital admissions: IRR: 1.04, 95% CI: 1.02, 1.06. The IRR of 1.04 was the same as in the complete case analysis.

The same process was repeated and we imputed missing data for all covariates and also for GAD. This was the effect estimate: IRR: 1.05, 95% CI: 1.03, 1.08.

2. Subsidiary analysis: association between GAD-MDD comorbidity and non-psychiatric hospital admissions

When different forms of GAD were considered in relation to health service use, the effect estimate for GAD-MDD comorbidity was the only one that emerged as statistically significant in the complete case analysis. Therefore, we repeated this analysis using multiple imputations for missing data (similar process to that described in the first instance – we imputed missing data for all covariates except for GAD and MDD) and obtained the following result: IRR=1.20, 95% CI: 1.18, 1.22. The IRR is very similar to that obtained in the complete case analysis.

For peer review only

Auxiliary variables used in the imputation models

Variable	Questionnaire	Description of variable
**Sociodemographic factors**		
History of psychiatric illness	Health and Lifestyle Questionnaire (HLQ)	Self-reported history of other psychiatric illness
History of back pain	Health and Lifestyle Questionnaire (HLQ)	Self-reported history of back pain
History of cholesterol	Health and Lifestyle Questionnaire (HLQ)	Self-reported history of cholesterol
History of migraine	Health and Lifestyle Questionnaire (HLQ)	Self-reported history of migraine
History of tumour	Health and Lifestyle Questionnaire (HLQ)	Self-reported history of tumour

The questionnaire used for these variables have been previously described in the methods.

References

1. Berglund P, Heeringa S. Multiple imputation of missing data using SAS. Cary, NC: *SAS Institute Inc*, 2014.

For peer review only

Please see the article line numbers (column on the right) and the explanations provided.

STROBE Statement—checklist of items that should be included in reports of observational studies

	Item No	Recommendation	Line numbers within the article
**Title and abstract**	1	(*a*) Indicate the study's design with a commonly used term in the title or the abstract	9, 44
		(*b*) Provide in the abstract an informative and balanced summary of what was done and what was found	35-70
**Introduction**			
Background/rationale	2	Explain the scientific background and rationale for the investigation being reported	95-142
Objectives	3	State specific objectives, including any prespecified hypotheses	144-148
**Methods**			
Study design	4	Present key elements of study design early in the paper	156
Setting	5	Describe the setting, locations, and relevant dates, including periods of recruitment, exposure, follow-up, and data collection	155-166, 228-229
Participants	6	(*a*) *Cohort study*—Give the eligibility criteria, and the sources and methods of selection of participants. Describe methods of follow-up<br>*Case-control study*—Give the eligibility criteria, and the sources and methods of case ascertainment and control selection. Give the rationale for the choice of cases and controls<br>*Cross-sectional study*—Give the eligibility criteria, and the sources and methods of selection of participants	155-158, 160-162, 164-166, and especially 168-170
		(*b*) *Cohort study*—For matched studies, give matching criteria and number of exposed and unexposed<br>*Case-control study*—For matched studies, give matching criteria and the number of controls per case	
Variables	7	Clearly define all outcomes, exposures, predictors, potential confounders, and effect modifiers. Give diagnostic criteria, if applicable	Outcome: 223-246<br>Exposure: 172-182<br>Confounders: 183-221
Data sources/	8*	For each variable of interest, give sources of data and details of methods of	There were two variables of interest in this study:

For peer review only - http://bmjopen.bmj.com/site/about/guidelines.xhtml

measurement		assessment (measurement). Describe comparability of assessment methods if there is more than one group	health service use and generalized anxiety disorder (223-249, 172-182). The others are potential confounders – in the methods I list them all, indicate how they were assessed and mention that they were collected through the baseline, self-reported postal HLQ questionnaire.
Bias	9	Describe any efforts to address potential sources of bias	I conducted a complete case analysis, followed by multiple imputations of missing data.
Study size	10	Explain how the study size was arrived at	281-283
Quantitative variables	11	Explain how quantitative variables were handled in the analyses. If applicable, describe which groupings were chosen and why	How the variables were handled in analyses: 253-279<br><br>I mention that these groupings were based on the literature and provide the relevant citations.<br><br>How the outcome variable was created: 225-246<br>How GAD was created: 174-182<br>Covariates: 185-221; I mention that the final categorization of the variables was also done in accordance with previous literature and I cite the relevant studies.
Statistical methods	12	(*a*) Describe all statistical methods, including those used to control for confounding	251-279
		(*b*) Describe any methods used to examine subgroups and interactions	There were no subgroups or interactions examined. In addition to the main question (if past-year GAD leads to higher health service use), we also determined whether GAD frequency, chronicity, age at onset, and comorbidity with MDD are associated with health service use – this is explained in lines 272-279.
		(*c*) Explain how missing data were addressed	We did a complete-case analysis followed by multiple imputations for missing data.
		(*d*) *Cohort study*—If applicable, explain how loss to follow-up was addressed<br>*Case-control study*—If applicable, explain how matching of cases and controls was	Loss to follow-up was not a problem in this study. We were able to track down all participants using

For peer review only - http://bmjopen.bmj.com/site/about/guidelines.xhtml

addressed<br>*Cross-sectional study*—If applicable, describe analytical methods taking account of sampling strategy	various means, unless they expressed that they wished to be removed from the mailing list. We elaborate on this in the manuscript.
(*e*) Describe any sensitivity analyses	

For peer review only

For peer review only - http://bmjopen.bmj.com/site/about/guidelines.xhtml

**Results**			
Participants	13*	(a) Report numbers of individuals at each stage of study—eg numbers potentially eligible, examined for eligibility, confirmed eligible, included in the study, completing follow-up, and analysed	
		(b) Give reasons for non-participation at each stage	We do not have the reasons for non-participation, because these data were not collected when the study was initiated in 1993.
		(c) Consider use of a flow diagram	Flow diagram included in submission – figure 1.
Descriptive data	14*	(a) Give characteristics of study participants (eg demographic, clinical, social) and information on exposures and potential confounders	302-303, 321-325, 342-345, as well as, tables 1 and 2
		(b) Indicate number of participants with missing data for each variable of interest	See flow diagram.
		(c) *Cohort study*—Summarise follow-up time (eg, average and total amount)	299-300
Outcome data	15*	*Cohort study*—Report numbers of outcome events or summary measures over time	Table 2 provides the mean number of hospital admissions for those with/without GAD over the 9-year follow-up.
		Case-control study—Report numbers in each exposure category, or summary measures of exposure	
		Cross-sectional study—Report numbers of outcome events or summary measures	
Main results	16	(*a*) Give unadjusted estimates and, if applicable, confounder-adjusted estimates and their precision (eg, 95% confidence interval). Make clear which confounders were adjusted for and why they were included	Tables 3 and 4 contain unadjusted and progressively adjusted estimates. We also discussed the findings within the text, and provide odds ratios and 95% confidence intervals.<br><br>We included the confounders based on the literature – we mention this in the paper and cite relevant literature. As per strobe, we included this information in the methods section; and we omitted repeating this in the results section to reduce redundancy. However, if the editor would like us to repeat this information in the results, we are happy to do so.

For peer review only

		(*b*) Report category boundaries when continuous variables were categorized	The cut-offs for age, alcohol intake, and disability levels are provided (see also footnotes of tables).
		(*c*) If relevant, consider translating estimates of relative risk into absolute risk for a meaningful time period	
Other analyses	17	Report other analyses done—eg analyses of subgroups and interactions, and sensitivity analyses	The Results section contains the findings from the multiple imputations for missing data analysis – we also included further information on this in an appendix. Findings for GAD frequency, chronicity, age of onset, and comorbidity with MDD are reported in table 4.
**Discussion**			
Key results	18	Summarise key results with reference to study objectives	416-422
Limitations	19	Discuss limitations of the study, taking into account sources of potential bias or imprecision. Discuss both direction and magnitude of any potential bias	438-476
Interpretation	20	Give a cautious overall interpretation of results considering objectives, limitations, multiplicity of analyses, results from similar studies, and other relevant evidence	514-551 (We also have a section comparing our study results to those of others: 478-499, as well as a section on potential mechanisms explaining our findings: 501-512)
Generalisability	21	Discuss the generalisability (external validity) of the study results	471-473, 549-468
**Other information**			
Funding	22	Give the source of funding and the role of the funders for the present study and, if applicable, for the original study on which the present article is based	567-568, 584-586

*Give information separately for cases and controls in case-control studies and, if applicable, for exposed and unexposed groups in cohort and cross-sectional studies.

Note: An Explanation and Elaboration article discusses each checklist item and gives methodological background and published examples of transparent reporting. The STROBE checklist is best used in conjunction with this article (freely available on the Web sites of PLoS Medicine at http://www.plosmedicine.org/, Annals of Internal Medicine at http://www.annals.org/, and Epidemiology at http://www.epidem.com/). Information on the STROBE Initiative is available at www.strobe-statement.org.

For peer review only - http://bmjopen.bmj.com/site/about/guidelines.xhtml

BMJ Open

Generalized anxiety disorder and non-psychiatric hospital admissions: findings from a large, population cohort study

Journal:	*BMJ Open*
Manuscript ID	bmjopen-2017-018539.R2
Article Type:	Research
Date Submitted by the Author:	18-Oct-2017
Complete List of Authors:	Remes, Olivia; University of Cambridge, Public Health and Primary Care<br>Wainwright, Nicholas; University of Cambridge, UK, Public Health and Primary Care<br>Surtees, Paul; University of Cambridge, Department of Public Health and Primary Care<br>LaFortune, Louise; University of Cambridge, Institute of Public Health<br>Khaw, Kay-Tee; University of Cambridge, Department of Public Health and Primary Care<br>Brayne, Carol; University of Cambridge, Institute of Public Health
**Primary Subject Heading**:	Mental health
Secondary Subject Heading:	Health services research, Public health
Keywords:	EPIDEMIOLOGY, MENTAL HEALTH, PUBLIC HEALTH

SCHOLARONE™
Manuscripts

For peer review only

Generalized anxiety disorder and non-psychiatric hospital admissions: findings from a large, population cohort study

Olivia Remes[1], Nicholas WJ Wainwright[2], Paul Surtees[1], Louise Lafortune[1], Kay-Tee Khaw[3], Carol Brayne[1]

[1]Department of Public Health and Primary Care, University of Cambridge, Forvie Site, Robinson Way, Cambridge, UK CB2 0SR

[2](Retired) Department of Public Health and Primary Care, University of Cambridge, 2 Worts' Causeway, Cambridge, UK CB1 8RN

[3]Department of Public Health and Primary Care, University of Cambridge, 2 Worts' Causeway, Cambridge, UK CB1 8RN

Figures: 1; Tables: 4

Correspondence to: Olivia Remes or260@medschl.cam.ac.uk, telephone: 07776740550

For peer review only

ABSTRACT

OBJECTIVE

Generalized anxiety disorder (GAD) is the most common anxiety disorder in the general population, and has been associated with high economic and human burden. However, it has been neglected in the health services literature. The objective of this study will be to assess whether GAD leads to non-psychiatric hospital admissions using data from the European Prospective Investigation of Cancer-Norfolk. Other aims include determining whether early or late onset forms of the disorder, episode chronicity and frequency, and comorbidity with major depressive disorder (MDD) contribute to non-psychiatric hospital admissions.

DESIGN

Large, population study.

SETTING

UK population-based cohort.

PARTICIPANTS

30,445 people over the age of 40 were recruited through general practice registers in England. Of these, 20,919 completed a structured health and lifestyle questionnaire used to assess GAD. Anxiety was examined in 1996-2000, and health service use was captured between 1999/00 and 2009 through record linkage with large, administrative health databases. 17,939 participants had complete data on covariates.

MAIN OUTCOME MEASURE

Past-year GAD defined according to the Diagnostic and Statistical Manual of Mental Disorders, fourth edition.

RESULTS

In this study, 2.2% (393/17,939) respondents had GAD. Anxiety was not independently associated with non-psychiatric hospital admissions (IRR=1.04, 95% CI: 0.90, 1.20) over nine years. However, those whose anxiety was comorbid with depression showed a statistically significantly increased risk for non-psychiatric hospital admissions (IRR=1.23, 95% CI: 1.02, 1.49).

CONCLUSION

People with GAD and MDD comorbidity were at an increased risk for hospital admissions. Clinicians should consider that meeting criteria for a pure or individual disorder at one point in time, such as past-year GAD does not necessarily predict deleterious health outcomes; rather different forms of the disorder, such as comorbid cases might be of greater importance.

Key words: Anxiety, anxiety disorders, health services

For peer review only

ARTICLE SUMMARY

Strengths and limitations of this study

- We used a large, population-based sample of middle- and older-aged adults and adjusted for a range of important confounders, such as, sociodemographic factors and medical history.
- We used a structured, self-reported questionnaire to assess presence of past-year GAD, and participants were followed for 9 years.
- We examined health services through record linkage with large, administrative health databases.
- Those who participated in this study were somewhat less deprived and healthier than individuals living in other parts of England; therefore, our results may not generalize to people living in extremely deprived circumstances.

For peer review only

INTRODUCTION

Anxiety disorders[1] are the most common class of psychiatric disorders in the general population. The Global Burden of Disease study[2] estimated that anxiety disorders contribute to 26.8 million disability adjusted life years, and their annual direct cost is $42.3 billion[3]. Generalized anxiety disorder (GAD) is characterized by excessive, pervasive worry, and a number of additional symptoms, such as restlessness and muscle tension. It is a prevalent and disabling condition in adults, and can lead to serious impairment in social and occupational functioning.[4] GAD is associated with poor quality of life, impaired functioning and risk of suicide.[5-8] Across the anxiety disorders, this condition has been found to be the most debilitating.[5, 9] Although there is effective treatment for GAD, only a third of those affected receive any treatment.[8] This is because anxiety disorders are frequently under-recognized and mismanaged by clinicians in primary care, which is often the first point of contact for those with mental health problems.[10]

Although detection of anxiety in clinical settings is poor[11, 12] and the presence of undiagnosed mental health problems can contribute to further emotional distress in patients down the line[12], it could be that disorders such as GAD represent more than just psychological or worry-related symptoms. It may be that anxiety symptoms are masking underlying poor physical health or could be an early warning signal for future health problems that are not yet detectable by standard medical tests. Such problems cannot be simply resolved through psychological therapies or psychotropic medication.

Anxiety has been linked to hypothalamic-pituitary-adrenal (HPA)-axis dysregulation and inflammation, and this can lead to poor health.[9] A recent study of hospitalized patients[13] also showed that people with anxiety disorders had more co-morbid physical conditions, including cardiovascular diseases and their risk factors, compared to people without anxiety disorders. Conversely, anxiety could also represent a response to underlying medical illness, and physical illness can exacerbate anxiety; the possibility of a bidirectional relationship between anxiety and physical health should not be excluded.[14, 15] Compelling evidence from prospective studies, however, has shown that anxiety can

For peer review only

indeed increase the risk of serious chronic conditions, such as cancer[16] and coronary heart disease (CHD)[17].

When investigating the links between mental disorders and health outcomes, early or late-onset forms of anxiety disorders, as well as psychiatric comorbidity should be also considered. A study[18] of over one million Swedish men followed for over 20 years showed that early-onset forms of mental disorders in particular led to increased risk of incident CHD. Anxiety disorders, such as, GAD are also frequently comorbid with major depressive disorder (MDD)[19], and psychiatric comorbidity has been associated with poorer quality of life, worse prognosis, and higher use of health services for mental health problems than pure forms of the disorder.[20, 21, 22] Therefore, identifying clinical aspects, such as, early or late onset forms of the condition, episode chronicity and frequency, and comorbidity with MDD can lead to better clinical management and more accurate prediction of future disability and health service use.[23]

GAD is one of the most common anxiety disorders in the general population[24] and the primary care setting[25], and has been associated with high economic and human burden. However, it has been neglected in the health services literature, with the exception of some studies showing GAD to contribute to higher use of primary care services in primary care samples.[26-29] Clinical samples, however, have the potential for self-selection bias. Whether GAD leads to non-psychiatric hospital admissions is unknown.

The objective of this study will be to assess the association between GAD and non-psychiatric hospital admissions in a longitudinal, population cohort of over 18,000 British individuals followed for 9 years. The aim is also to determine whether early or late onset forms of the disorder, episode frequency and chronicity, and comorbidity with MDD contribute to non-psychiatric hospital admissions.

For peer review only

METHODS

Study population

The study population was drawn from the European Prospective Investigation of Cancer-Norfolk longitudinal, cohort study, described in detail elsewhere.[30, 31] Briefly, a total of 30,445 participants over the age of 40 living in Norwich and the surrounding towns and rural areas were recruited between 1993 and 1997 using general practice registers. At baseline, they completed a health questionnaire capturing sociodemographics and medical history. During follow-up, between 1993 and 1999/2000, participants completed self-reported postal questionnaires provided they: 1) were still alive, 2) did not ask to be removed from the study's mailing list, and 3) had a valid mailing address. Between 1996 and 1999/2000, respondents completed a Health and Life Experiences Questionnaire (HLEQ)[30] used to capture information on psychiatric disorders, other psychosocial factors, and risk behaviours. Record linkage with administrative health databases using a unique identifier was used to determine hospitalization admissions data until 2009.

All participants recruited through general-practice registers and who completed a baseline health questionnaire were eligible to be included in our study; those who completed a psychosocial questionnaire during follow-up were eligible to be included in our analysis.

Assessment of GAD

The HLEQ was used to derive a measure of GAD according to the Diagnostic and Statistical Manual of Mental Disorders, fourth edition. The HLEQ captured the onset and offset timings of episodes of past-year GAD.[32] Past-year GAD consisted of at least one episode that had offset within 12 months of administration of the HLEQ. DSM-IV GAD was diagnosed if participants reported having uncontrollable, excessive worry for six months or longer on most days than not that resulted in disability or impairment. In addition, at least three of the following symptoms needed to have been present: restlessness, irritability, muscle tension, fatigue, trouble concentrating because of worry, mind going blank, trouble falling asleep, trouble staying asleep, and feeling keyed up or on edge.

For peer review only

Assessment of covariates

Potential confounders (based on the literature) included sociodemographics (age, sex, education, marital status, social class, employment), prevalent physical diseases, disability, MDD, and risk behaviours (alcohol use, smoking, physical activity). The final categorization of the variables took cell size into account and was also done in accordance with previous literature.[32, 33-38] Age was first assessed as a categorical variable, and subsequently divided into 10-year bands. Sex was categorized into male vs. female; marital status was categorized into: married, single (or never married), and others (widowed, divorced, separated); educational attainment into high (vocational or formal qualifications at the A- or O-level or degree-level qualifications) vs. low (no formal qualifications). Social class was derived using the Computer-Assisted Standard Occupational Coding[38] and categorized as follows: I (professionals), II (managerial and technical occupations), III non-manual and III manual (skilled workers), IV (partly skilled workers), and V (unskilled manual workers). For men, social class was coded using their own occupation except when they were unemployed or retired in which case their partner‘s social class was used. Unemployed men without partners were unclassified. Social class in women was based on their partner‘s except when the partner‘s social class was unclassified, missing, or they had no partner in which case social class was based on their own occupation. An unemployed woman without a partner was coded as unclassified. The final categorization of social class included manual: skilled manual, partly skilled, and unskilled; and non-manual: professionals, managerial and technical, and skilled non-manual. Employment was divided into yes vs. no.

Behaviour risk factor measures included alcohol intake (units of alcohol/week), smoking status (current, former, non-smoker), and physical activity (inactive, moderately inactive, moderately active, active). Presence of past-year DSM-IV major depressive disorder (MDD) (yes/no) was also assessed.[39]

Individual-level health status was assessed through the construction of a variable capturing major prevalent physical diseases associated with anxiety.[40] This was based on HLQ questions asking participants: “Has the doctor ever told you that you have any of the following?”, followed by a list of options, such as stroke, myocardial infarction, and cancer.

For peer review only

To determine disability levels, we used the physical component summary score (PCS) of the Medical Outcomes Study 36-Item Short Form (SF-36), a widely-used, validated self-assessment tool. A score of 100 represents no disability and 0 represents a high level of disability.[41] PCS scores were dichotomized above and below the median.

All of these individual-level variables were regarded as potential confounders and selected based on the literature and their association with anxiety and health service use.[42, 43]

Hospital service use

All analyses are based on non-psychiatric hospitalizations. Primary care service use was not captured in this study.

Frequency of hospitalization between 1999/00 and 2009 was determined using administrative health databases maintained by the National Health Service. The East Norfolk Primary Health Care trust databases were used, and these are updated on an ongoing basis and provide information on clinical and administrative data from participating facilities, such as, hospitals.

England is under a publicly-funded health care system (the National Health Service), free at the point of delivery; therefore, we expect factors, such as access to health insurance or personal income, to have minimal impact on the care that is obtained by study participants. The databases used in this study are maintained by the National Health Service, which is likely to capture most hospital admissions from the population, as private sector provision is minimal. This means that admissions data in our study can be considered complete for the ascertainment of hospital/health service use, and the likelihood of bias minimal. To access hospital services in the UK, a referral is needed from the primary care practitioner, who acts as a gate-keeper to secondary care.

The East Norfolk Primary Health Care databases were linked to the EPIC-Norfolk cohort using participants' unique National Health Service number, which allows complete record linkage across settings and calendar time.

For peer review only

Vital status for participants was determined through record linkage with the United Kingdom Office of National Statistics. Vital status was available for all participants. This allowed us to exclude those who died before their health service use was ascertained.

Statistical Analysis

First, demographics, social class, medical and psychiatric conditions, and risk behaviours were compared by GAD status - the chi-square test was used to determine whether differences were statistically significant for categorical variables. Second, the mean number of hospital admissions was determined for each characteristic/covariate - the Kruskal Wallis test was used to determine statistical significance for categorical covariates with three or more categories, while the Wilcoxon rank-sum test was used for dichotomous covariates.

Since the number of hospital admissions was skewed and the variance was much larger than the mean, zero-inflated negative binomial regression was used for frequency of hospital utilization (number of hospital admissions). The log-likelihood test showed that this model was superior to Poisson regression. Three models were fitted for hospital admissions with progressive adjustment of covariates: model A adjusted for sociodemographics (age, sex, education, marital status, social class, employment), physical conditions and disability; model B further accounted for past-year MDD (assessed at the same questionnaire point as past-year GAD); and model C further controlled for physical activity, alcohol, and smoking. Multiple imputations for missing data were also carried out on the fully-adjusted model assessing the association between past-year GAD and non-psychiatric hospitalization (our primary objective).

Finally, we determined whether the risk for hospitalization was higher among those with: 1) 3 or more episodes of lifetime GAD (versus those with fewer than 3 episodes or no GAD), 2) episodes that lasted on average 6 months or more (versus those with fewer than 6 months or no GAD), 3) age of onset at 30 years or younger (versus people with age at onset over 30 years or no GAD), and 4) psychiatric comorbidity with MDD (versus no GAD-MDD comorbidity). Two-sided statistical tests for the maximum likelihood zero inflation

For peer review only

parameter estimates were conducted and a p-value of <0.05 was used for statistical significance. Analyses were implemented in SAS, version 9.3.

To arrive at the study size, we went through the following steps: of the 30,445 who completed the baseline HLQ, we retained those participants who completed the HLEQ (20,919), and of these, we kept those people with complete data on all covariates (17,939). (Figure 1)

Patient involvement

There were no patients involved in the development of the research question and outcome measures, the design of the study, or the recruitment to and conduct of the study.

For peer review only

RESULTS

Of the 30,445 people recruited at baseline, 20,919 participants completed the HLEQ; most of the missing observations were from past-year GAD (479), past-year MDD (700), and disability (1386); the rest of the missing observations were generated from the other covariates (Figure 1). Notable findings from the missing data analysis show that people with missing GAD more often had pre-existing health conditions, high disability, MDD, low alcohol consumption, and were without employment (Appendix I).

The final sample included a total of 17,939 participants. Participants were assessed between 1999/00 and 2009 (followed for 9 years) (Figure 1).

In 1996-2000, GAD was present in 393 out of 17,939 (2.2%) people. Table 1 shows the baseline characteristics of participants by GAD status.

For peer review only

Table 1 Percentage and number of people with past-year GAD reported in 1996-2000 according to sociodemographic factors, health status, and behaviour risk factors for the EPIC-Norfolk cohort (n=17,939)

Characteristic	Number with characteristic	Percentage and number with past-year GAD
**Socio-demographics**		
**Age (years)**		
<50	2359	3.4 (79)[a]
50-60	6209	2.9 (179)
60-70	5733	1.6 (94)
70+	3638	1.1 (41)
**Sex**		
Women	9937	2.5 (249)[b]
Men	8002	1.8 (144)
**Education**[‡]		
Low	6106	2.0 (120)[b]
High	11833	2.3 (273)
**Marital status**		
Single	686	3.6 (25)[a]
Married	14538	2.0 (284)
Other[*]	2715	3.1 (84)
**Social class**		
Manual	6836	2.0 (137)
Non-manual	11103	2.3 (256)
**Employment**		
Yes	7712	2.0 (155)
No	10227	2.3 (238)
**Health status**		
**Physical conditions**[+]		
Yes	9166	2.7 (251)[a]
No	8773	1.6 (142)
**Disability level**		
High[¶]	8900	3.0 (266)[a]
Low	9039	1.4 (127)
**Psychiatric conditions**		
**Past-year MDD**		
Yes	934	21.4 (200)[a]
No	17005	1.1 (193)
**Behaviour risk factors**		
**Physical activity**		
Active[¥]	12822	2.1 (272)
Inactive	5117	2.4 (121)
**Smoking status**		
Current smoker	1893	4.7 (89)[a]
Former smoker	7470	1.9 (141)
Never smoker	8576	1.9 (163)
**Alcohol intake**		
High[α]	9241	2.0 (182)[b]

For peer review only

Low	8698	2.4 (211)

‡ High education: O-level, A-level, degree; low education: refers to no education

* Other: divorced, separated, widowed

+ Physical conditions: respiratory disease (asthma and bronchitis), allergies and hay fever, stroke, heart attack, cancer, diabetes, thyroid conditions, arthritis

¶ Below the median PCS value of 50.6

¥ Moderately inactive, moderately active, active

α 3+ units of alc./week (1 pint beer=2 units, 1 glass wine=1 unit, 1 glass sherry=1 unit, 1 glass spirits=1 unit)

[a] *P* < 0.001

[b] *P* < 0.05

For peer review only

Those with GAD were more likely to be younger, women, inactive, current smokers, low alcohol consumers, of higher educational attainment, single, of non-manual social class, without employment, with physical conditions, high levels of disability, and MDD. Table 2 summarizes the means and standard deviations of the number of hospital admissions by participant characteristics.

For peer review only

Table 2: Non-psychiatric hospital admissions (mean, SD) by participant characteristics in 17,939 British people between 1999/00 and 2009

	Total number with characteristic	Number of admissions
**Characteristic**		**Mean (SD)**
**Past-year GAD**		
Yes	393	4.0 (6.3)[a]
No	17546	3.4 (13.0)
**Socio-demographics**		
**Age (years)**		
<50	2359	1.9 (9.8)[a]
50-60	6209	3.0 (16.5)
60-70	5733	3.8 (11.2)
70+	3638	4.6 (9.6)
**Sex**		
Women	9937	3.1 (14.0)[a]
Men	8002	3.9 (11.3)
**Education**[‡]		
Low	6106	4.1 (17.1)[a]
High	11833	3.1 (10.1)
**Marital status**		
Single	686	3.0 (8.9)[a]
Married	14538	3.3 (10.9)
Other[*]	2715	4.0 (21.0)
**Social class**		
Manual	6836	4.0 (18.3)[a]
Non-manual	11103	3.1 (7.8)
**Employment**		
Yes	7712	2.5 (9.1)[a]
No	10227	4.1 (15.1)
**Health status**		
**Physical conditions**[+]		
Yes	9166	3.9 (10.4)[a]
No	8773	3.0 (15.1)
**Disability level**		
High[¶]	8900	4.4 (16.5)[a]
Low	9039	2.5 (7.8)
**Psychiatric conditions**		
**Past-year MDD**		
Yes	934	4.5 (13.6)[a]
No	17005	3.4 (12.9)
**Behaviour risk factors**		
**Physical activity**		
Active[¥]	12822	3.2 (13.3)[a]
Inactive	5117	4.1 (11.7)
**Smoking status**		
Current smoker	1893	4.6 (26.8)[a]

For peer review only

Former smoker	7470	3.8 (11.4)
Never smoker	8576	2.9 (8.6)
**Alcohol intake**		
High[α]	9241	3.2 (13.3)[a]
Low	8698	3.7 (12.5)

‡ High education: O-level, A-level, degree; low education: refers to no education

* Other: divorced, separated, widowed

+ Physical conditions: respiratory disease (asthma and bronchitis), allergies and hay fever, stroke, heart attack, cancer, diabetes, thyroid conditions, arthritis

¶ Below the median PCS value of 50.6

¥ Moderately inactive, moderately active, active

α 3+ units of alc./week (1 pint beer=2 units, 1 glass wine=1 unit, 1 glass sherry=1 unit, 1 glass spirits=1 unit)

[a] $P < 0.001$

[b] $P < 0.05$

For peer review only

Participants with GAD had a higher frequency of hospitalization compared to those without GAD. Some of the findings show that frequency of hospitalization was markedly higher among older age groups, men, those with low educational attainment, unemployed participants, those with high levels of disability, and with past-year MDD.

Table 3 shows the unadjusted and adjusted incidence rate ratios of hospital admissions by GAD status.

For peer review only

Table 3: Associations between past-year GAD reported in 1996-2000 and non-psychiatric hospital admissions in 1999/00-2009 in 17,939 British people over the age of 40

	IRR and 95% CI			
**Characteristic**	**Crude IRR**	**A[1]**	**B[2]**	**C[3]**
**Past-year GAD**				
Yes	1.18 (1.02, 1.36)	1.25 (1.09, 1.43)	1.10 (0.96, 1.27)	1.04 (0.90, 1.20)
No	1.00	1.00	1.00	1.00
**Socio-demographics**				
**Age**				
Per 10 years	1.36 (1.33, 1.40)	1.19 (1.16, 1.23)	1.20 (1.17, 1.24)	1.21 (1.18, 1.25)
**Sex**				
Women	0.80 (0.76, 0.83)	0.76 (0.73, 0.79)	0.76 (0.72, 0.79)	0.78 (0.74, 0.81)
Men	1.00	1.00	1.00	1.00
**Education[‡]**				
Low	1.30 (1.24, 1.36)	1.13 (1.08, 1.18)	1.13 (1.08, 1.19)	1.11 (1.06, 1.16)
High	1.00	1.00	1.00	1.00
**Marital status**				
Single	0.88 (0.79, 0.99)	0.85 (0.77, 0.95)	0.85 (0.76, 0.95)	0.84 (0.76, 0.94)
Married	1.00	1.00	1.00	1.00
Other[*]	1.21 (1.14, 1.28)	1.17 (1.11, 1.24)	1.14 (1.07, 1.21)	1.09 (1.03, 1.16)
**Social class**				
Manual	1.29 (1.23, 1.34)	1.24 (1.19, 1.30)	1.24 (1.19, 1.30)	1.21 (1.16, 1.26)
Non-manual	1.00	1.00	1.00	1.00
**Employment**				
Yes	1.00	1.00	1.00	1.00
No	1.64 (1.57, 1.71)	1.18 (1.12, 1.25)	1.18 (1.12, 1.24)	1.15 (1.09, 1.21)
**Health status**				
**Physical conditions[+]**				
Yes	1.32 (1.26, 1.37)	1.18 (1.13, 1.23)	1.17 (1.12, 1.22)	1.18 (1.13, 1.23)
No	1.00	1.00	1.00	1.00
**Disability level**				
High[¶]	1.78 (1.71, 1.86)	1.52 (1.45, 1.59)	1.51 (1.44, 1.57)	1.48 (1.42, 1.55)
Low	1.00	1.00	1.00	1.00
**Psychiatric conditions**				
**Past-year MDD**				
Yes	1.34 (1.22, 1.48)		1.34 (1.22, 1.48)	1.33 (1.21, 1.46)
No	1.00		1.00	1.00
**Lifestyle**				
**Physical activity**				
Active[¥]	1.00			1.00
Inactive	1.27 (1.21, 1.33)			1.04 (1.00, 1.09)
**Smoking status**				
Current smoker	1.60 (1.49, 1.72)			1.51 (1.41, 1.62)
Former smoker	1.33 (1.27, 1.39)			1.13 (1.08, 1.18)

For peer review only - http://bmjopen.bmj.com/site/about/guidelines.xhtml

Never smoker	1.00		1.00
**Alcohol intake**			
High[α]	0.88 (0.85, 0.92)		0.92 (0.88, 0.96)
Low	1.00		1.00

[1] Model A: adjusted for sociodemographics (age, sex, education, marital status, social class, employment), physical conditions, disability

[2] Model B: adjusted for sociodemographics, physical conditions, disability, MDD

[3] Model C: adjusted for sociodemographics, physical conditions, disability, MDD, physical activity, smoking, alcohol

‡ High education: O-level, A-level, degree; low education: refers to no education

* Other: divorced, separated, widowed

+ Physical conditions: respiratory disease (asthma and bronchitis), allergies and hay fever, stroke, heart attack, cancer, diabetes, thyroid conditions, arthritis

¶ Below the median PCS value of 50.6

¥ Moderately inactive, moderately active, active

α 3+ units of alc./week (1 pint beer=2 units, 1 glass wine=1 unit, 1 glass sherry=1 unit, 1 glass spirits=1 unit)

For peer review only

After adjustment for sociodemographic variables, physical conditions, and disability, GAD was associated with a 25% higher incidence rate of hospitalization (IRR=1.25, 95% CI: 1.09, 1.43). The incidence rate ratio was somewhat attenuated and became statistically non-significant after further adjustment for MDD (IRR=1.10, 95% CI 0.96, 1.27). The effect estimate approached the null after additional adjustment for behaviour risk factors (IRR=1.04, 95% CI: 0.90, 1.20). Finally, we did multiple imputations for missing data (Appendix II). The effect estimate remained the same when we imputed all covariates except for GAD (IRR: 1.04, 95% CI: 1.02, 1.06). The effect estimate remained virtually unchanged when we imputed all covariates including GAD (IRR: 1.05, 95% CI: 1.03, 1.08).

Next, we assessed whether risk for hospital admissions varied by frequency of GAD lifetime episodes, anxiety episode chronicity, GAD age of onset, and whether the hospitalization risk was higher in those with psychiatric comorbidity (with MDD) (table 4). Results are based on fully-adjusted models.

For peer review only

Table 4: Associations between different forms of GAD reported in 1996-2000 and non-psychiatric hospital admissions in 1999/00-2009 in 17,939 British people ages 40+

Characteristic	IRR and 95% CI			
**GAD type**				
**Frequent GAD**				
Yes[a]	1.07 (0.91, 1.27)[e]			
No	1.00			
**Chronic GAD**				
Yes[b]		1.07 (0.85, 1.35)[e]		
No		1.00		
**Early age GAD onset**				
Yes[c]			1.16 (0.95, 1.41)[e]	
No			1.00	
**Comorbid GAD**				1.23 (1.02, 1.49)[e]
Yes[d]				1.00
No				
**Socio-demographics**				
**Age**				
Per 10 years	1.22 (1.18, 1.25)	1.21 (1.18, 1.25)	1.22 (1.18, 1.25)	1.21 (1.17, 1.25)
**Sex**				
Women	0.78 (0.74, 0.81)	0.78 (0.74, 0.81)	0.78 (0.74, 0.81)	0.78 (0.75, 0.82)
Men	1.00	1.00	1.00	1.00
**Education**[‡]				
Low	1.10 (1.06, 1.16)	1.11 (1.06, 1.16)	1.10 (1.06, 1.16)	1.12 (1.07, 1.18)
High	1.00	1.00	1.00	1.00
**Marital status**				
Single	0.84 (0.76, 0.94)	0.84 (0.76, 0.94)	0.84 (0.76, 0.94)	0.83 (0.74, 0.93)
Married	1.00	1.00	1.00	1.00
Other[*]	1.10 (1.03, 1.16)	1.09 (1.03, 1.16)	1.09 (1.03, 1.16)	1.03 (0.97, 1.10)
**Social class**				
Manual	1.21 (1.16, 1.26)	1.21 (1.16, 1.26)	1.21 (1.16, 1.26)	1.21 (1.16, 1.27)
Non-manual	1.00	1.00	1.00	1.00
**Employment**				
Yes	1.00	1.00	1.00	1.00
No	1.15 (1.09, 1.21)	1.15 (1.09, 1.21)	1.15 (1.09, 1.21)	1.17 (1.11, 1.24)
**Health status**				
**Physical conditions**[+]				
Yes	1.17 (1.13, 1.23)	1.17 (1.13, 1.23)	1.17 (1.13, 1.23)	1.17 (1.12, 1.22)
No	1.00	1.00	1.00	1.00
**Disability level**				
High[¶]	1.48 (1.42, 1.55)	1.48 (1.42, 1.55)	1.48 (1.42, 1.55)	1.48 (1.42, 1.55)
Low	1.00	1.00	1.00	1.00
**Psychiatric conditions**				

For peer review only

**Past-year MDD**				
Yes	1.32 (1.20, 1.45)	1.33 (1.22, 1.46)	1.33 (1.21, 1.46)	--
No	1.00	1.00	1.00	
**Lifestyle**				
**Physical activity**				
Active[¥]	1.00	1.00	1.00	1.02 (0.97, 1.07)
Inactive	1.04 (1.00, 1.09)	1.04 (1.00, 1.09)	1.04 (1.00, 1.09)	1.00
**Smoking status**				
Current smoker	1.51 (1.41, 1.62)	1.51 (1.41, 1.62)	1.51 (1.41, 1.62)	1.56 (1.45, 1.68)
Former smoker	1.13 (1.08, 1.18)	1.13 (1.08, 1.18)	1.13 (1.08, 1.18)	1.14 (1.09, 1.19)
Never smoker	1.00	1.00	1.00	1.00
**Alcohol intake**				
High[α]	0.92 (0.88, 0.96)	0.92 (0.88, 0.96)	0.92 (0.88, 0.96)	0.93 (0.89, 0.98)
Low	1.00	1.00	1.00	1.00

[a] 3+ episodes of lifetime GAD
[b] GAD episodes lasted at least 6 months
[c] GAD developed before 30 years of age
[d] GAD-MDD comorbidity
[e] Adjusted for sociodemographics, physical conditions, disability, MDD, physical activity, smoking, alcohol

[‡] High education: O-level, A-level, degree; low education: refers to no education
[*] Other: divorced, separated, widowed
[+] Physical conditions: respiratory disease (asthma and bronchitis), allergies and hay fever, stroke, heart attack, cancer, diabetes, thyroid conditions, arthritis
[¶] Below the median PCS value of 50.6
[¥] Moderately inactive, moderately active, active
[α] 3+ units of alc./week (1 pint beer=2 units, 1 glass wine=1 unit, 1 glass sherry=1 unit, 1 glass spirits=1 unit)

For peer review only

People with more than 3 lifetime episodes had a somewhat higher risk of hospitalization (IRR=1.07, 95% CI: 0.91, 1.27). Those whose episodes lasted, on average, 6 months or longer also had a slight increased risk for admissions compared to those with shorter episodes (IRR=1.07, 95% CI: 0.85, 1.35). People who developed GAD before 30 years of age were 16% more likely to be admitted to the hospital than those who developed it later in life (IRR=1.16, 95% CI: 0.95, 1.41), although this finding was not statistically significant. Finally, we determined whether GAD comorbid with MDD is associated with non-psychiatric hospital admissions. Results showed that people with GAD-MDD comorbidity had a 23% higher chance of being admitted to hospital than people without comorbidity – this association was statistically significant (IRR: 1.23 ,95% CI: 1.02, 1.49).

For peer review only

DISCUSSION

This is the first study to assess the association between GAD and hospital service use in a population-based cohort. This longitudinal study showed that having an episode of GAD in the past year was not independently associated with hospital admissions during the subsequent nine years. Chronic GAD (at least 6 months), frequent GAD (at least 3 lifetime episodes), and anxiety with an early age of onset (before 30 years) did not show statistically significant associations with non-psychiatric hospitalizations. In contrast, people with GAD and MDD comorbidity were at an increased risk of being admitted to hospital than those without MDD comorbidity. The association between GAD-MDD comorbidity and non-psychiatric hospital admissions was statistically significant.

People with past-year GAD were more likely to have medical conditions; nonetheless, including these covariates in the model left the association between past-year GAD and hospital admissions statistically significant. It was only when MDD was introduced in the model as a potential confounder that any remaining association with hospital service utilization was explained away.

Strengths and limitations

There are several strengths associated with our study. We had a large, population-based sample of middle- and older-aged adults and adequately adjusted for a range of possible confounders. We used a structured questionnaire to assess past-year GAD according to DSM-IV criteria, used large administrative health databases to examine hospital service use (avoiding the self-reporting bias found in questionnaire studies), and participants were followed for a long time. We had a large list of self-reported physician diagnoses of chronic diseases that we used to ascertain medical histories. Despite this, the residual effect of diseases not captured by our study, but that are associated with GAD may be present. Past illness may have been underreported, which may have introduced measurement error and further attenuated effect estimates towards the null. A negligible proportion of participants may have obtained care at private facilities, which might have led to non-differential misclassification. The databases used in this study also did not capture admissions to

For peer review only

hospitals outside the UK. However, migration in the EPIC-Norfolk cohort is minimal and does not present a problem.

We may have overadjusted our models with the inclusion of self-evaluated impairment, as this may be part of the expression of psychiatric illness. This can lead to attenuation of effect estimates. If participants chose not to answer certain questions in the HLEQ, this contributed to missing data; however, to avoid biasing the findings, we retained participants for whom we had complete data on all covariates. Non-participation in our study also may have led to non-differential misclassification and further attenuation of effect estimates.

Another limitation is that we did not have data on primary care service use. Merging population cohorts, such as ours, with primary care service administrative databases and hospitalization databases would have provided a more complete picture of the burden of GAD on the health care system.

This study was conducted on people ages 40 years and older and may not be generalizable to younger age groups. We suspect that the strength of the association between GAD-MDD comorbidity and non-psychiatric hospital admissions is weaker for younger populations who are typically healthier than older people. Although young people have a high burden of mental health problems[40, 44], they (especially adolescents) are less likely to have non-psychiatric hospitalizations than older people[45]. It could take many years until the effects of anxiety comorbid with depression accumulate and manifest as poor physical health, thus translating into higher use of non-psychiatric hospital services. As such, we would expect the strength of the association between GAD-MDD comorbidity and hospitalizations to be weaker in young people, however, future studies should investigate this.

Participants were required to complete detailed dietary and lifestyle questionnaires and undergo periodic health assessments. Because those who participated in EPIC-Norfolk were more affluent and healthier than individuals living in other parts of England, our results may not generalize to people living in extremely deprived areas.

For peer review only

Finally, there was missing data in this study. When we conducted multiple imputations for missing data, the effect estimate of our main analysis remained unchanged.

Comparison with other studies

Most of the studies assessing the link between psychiatric disorders and non-psychiatric health service utilization have focused on depression and, to a lesser extent, panic disorder and PTSD, while other anxiety disorders have been significantly underresearched. Most of the studies on depression as a stand alone measure have shown an association with health service use in both clinical and community samples.[46] There are substantially fewer studies on anxiety, and a number of these have shown positive associations with health service use. A US study[47] that recruited patients from an outpatient clinic showed that anxiety disorders were linked to higher utilization of primary care services compared to depressive or addictive disorders. Patients, however, were recruited from an outpatient clinic located in a predominantly rural area, which might have affected generalizability. Another study showed anxiety disorders to be associated with a higher number of consultations in general medical, emergency and specialty settings, such as cardiology and dermatology.[48] In this study, people were sampled from an anxiety clinic, thereby leading to possible selection bias. Other studies showed PTSD and GAD to be associated with health care use, however, this research was based on highly-select samples that have limited generalizability.[26, 28, 49, 50] In contrast to the literature, a major strength of our study was that it was population-based. There is also a lack of research assessing whether different forms of the disorder contribute to even higher health service use rates (comorbid cases are typically the most severe, hardest to treat and with the poorest prognosis [9]).

Mechanisms

A more severe course of GAD can lead to higher rates of health services because of unhealthy behaviours, such as smoking and alcohol (which we controlled for in our analyses). It could also be that a more severe form of anxiety, such as GAD-MDD comorbidity is associated with poorer underlying health, which then leads to higher health service use rates. Although we controlled for several chronic diseases, we might have

For peer review only

missed some conditions that are associated with GAD-MDD comorbidity and hospitalizations. A third explanation for higher health service use in those with comorbid anxiety and depression could relate to inflammatory pathways. If clinically apparent signs of disease have not yet developed in those with psychiatric comorbidity or are at an early, undetectable stage, it will not be possible to measure these factors and adjust for them in analyses.

Implications

GAD is a debilitating and impairing condition.[9] The evidence base on its association with health services is small and confined to clinical settings with the potential for self-selection bias. Our study overcomes many limitations of previous studies, and clarifies that individual episodes of GAD measured at a single point in time (ex. in the past year) are not associated with health service use. Instead, it shows that cases that are comorbid with depression can lead to increased use of hospital services, after controlling for a range of important confounders. In this study, GAD-MDD comorbidity was associated with a statistically significantly increased risk of hospital admissions.

Population-based research on anxiety is lacking, and thus far, no studies have assessed the association between GAD and non-psychiatric hospitalization. Clinicians should consider that it is not just the diagnosis of the individual disorder at one point in time (ex. past-year GAD) that is predictive of deleterious health outcomes; different forms of the disorder may also be important. GAD has a waxing and waning course throughout a patient's life, and many of those affected experience relapse after psychiatric treatment or develop psychiatric comorbidities.

Our findings are important for clinicians and policy-makers. Large numbers of people are affected by anxiety-depression comorbidity [9]. As such, clinicians should consider more widespread screening for mental health problems and if appropriate, the examination of any underlying health conditions that may require treatment in order to prevent future hospital admissions. Policy-makers should also consider rolling out more widespread anxiety and depression prevention and screening programmes.

For peer review only

Future research, however needs to examine the reasons for the increased non-psychiatric hospital service use in those with GAD-MDD comorbidity (this can provide additional insight into clinical recommendations). To provide a better understanding of the links between mental and physical health, the bidirectional links between anxiety and physical health problems should also be examined. Finally, future research should merge a population-based cohort with primary and secondary care administrative health databases to provide a more complete picture of the burden of different forms of anxiety on the health care system.

Conclusion

People with GAD that was comorbid with MDD had a higher risk for hospital admissions over 9 years between 1996-1999/00 and 2009 in the European Prospective Investigation of Cancer in Norfolk study.

For peer review only

Acknowledgements: OR received a PhD studentship from the National Institute for Health Research.

Competing interest: All authors have completed the ICMJE uniform disclosure form at www.icmje.org/coi_disclosure.pdf and declare: no support from any organisation for the submitted work; no financial relationships with any organizations that might have an interest in the submitted work in the previous three years; no other relationships or activities that could appear to have influenced the submitted work.

Funding: This work was supported by the Medical Research Council UK (grant number SP2024-0201 and SP2024-0204) and Cancer Research UK (grant number G9502233).

Author contributions: OR (corresponding author) had the idea for and conducted the analysis, and wrote the article. CB critically reviewed drafts of the manuscript, KK edited versions of the paper; PS and NW provided feedback into the analysis. OR, CB, KK, LL, PS, and NW contributed to the interpretation of data for the work, agreed to be accountable for all aspects of the work, gave final approval of the version to be published, and made substantial contributions to the analysis and interpretation of data. OR, CB, KK, LL, PS, and NW have seen and approved the final version. OR, CB, KK, LL, PS, and NW had full access to all the data in the study and take responsibility for the integrity of the data and the accuracy of the data analysis. OR acts as guarantor of the study.

Transparency declaration: OR affirms that the manuscript is an honest, accurate, and transparent account of the study being reported; that no important aspects of the study have been omitted; and that any discrepancies from the study as planned have been explained.

Role of study sponsors and statement of independence: The funding sources had no role in the design and conduct of the study; collection, management, analysis, and interpretation of the data; and preparation, review, or approval of the manuscript.

For peer review only

Ethical approval: The study has ethics committee approval from Norfolk Ethics Committee (Rec Ref: 98CN01) and all participants gave informed consent.

Data sharing: No additional data available. Original dataset requests should be sent to the corresponding author. Please contact O Remes at or260@medschl.cam.ac.uk for questions about the statistical code.

Copyright: “The Corresponding Author has the right to grant on behalf of all authors and does grant on behalf of all authors, a worldwide licence to the Publishers and its licensees in perpetuity, in all forms, formats and media (whether known now or created in the future), to i) publish, reproduce, distribute, display and store the Contribution, ii) translate the Contribution into other languages, create adaptations, reprints, include within collections and create summaries, extracts and/or, abstracts of the Contribution, iii) create any other derivative work(s) based on the Contribution, iv) to exploit all subsidiary rights in the Contribution, v) the inclusion of electronic links from the Contribution to third party material where-ever it may be located; and, vi) licence any third party to do any or all of the above.”

For peer review only

REFERENCES

1. American Psychiatric Association. (1994). *Diagnostic and Statistical Manual of Mental Disorders* (4th edn). Washington, DC: APA.
2. Whiteford HA, Degenhardt, Rehm J, et al. Global burden of disease attributable to mental and substance use disorders: findings from the global burden of disease study 2010. *Lancet* 2013;13:1575-86 doi: http://dx.doi.org/10.1016/S0140-6736(13)61611-6.
3. Greenberg PE, Sisitsky T, Kessler RC, et al. The economic burden of anxiety disorders in the 1990s. *J Clin Psychiatry* 1999;60:427-35.
4. Kroenke K, Spitzer RL, Williams JB, et al. Anxiety disorders in primary care: prevalence, impairment, comorbidity, and detection. *Ann Intern Med* 2007;146:317-25 doi: 10.7326/0003-4819-146-5-200703060-00004.
5. Comer JS, Blanco C, Hasin DS, et al. Health-related quality of life across the anxiety disorders. *J Clin Psychiatry* 2011;72:43–50 doi: 10.4088/JCP.09m05094blu [published Online First 24 August 2010].
6. Tyrer P, Baldwin D. Generalised anxiety disorder. *Lancet* 2006;16:2156–66 doi: http://dx.doi.org/10.1016/S0140-6736(06)69865-6.
7. Hoge E, Ivkovic A, Fricchione G. Generalized anxiety disorder: diagnosis and treatment. *BMJ* 2012;345:e7500 doi: https://doi.org/10.1136/bmj.e7500.
8. Revicki DA, Travers K, Wyrwich KW. Humanistic and economic burden of generalized anxiety disorder in North America and Europe. *J Affect Disord* 2012;140:103–12 doi: 10.1016/j.jad.2011.11.014 [published Online First 9 December 2011].
9. Simpson HB, Neria Y, Lewis-Fernandez R, et al. Anxiety disorders. Theory, research and clinical perspectives. New York, US: Cambridge University Press 2010.
10. Combs H, Markman J. Anxiety disorders in primary care. *Med Clin North Am* 2014;98:1007-23 doi: 10.1016/j.mcna.2014.06.003 [published Online First 11 July 2014].
11. Buszewicz MJ, Chew-Graham C. Improving the detection and management of anxiety disorders in primary care. *Br J Gen Pract* 2011;61:489-490 doi: 10.3399/bjgp11X588259.

For peer review only

12. Wittchen H-U, Muhlig S, Beesdo K. Mental disorders in primary care. *Dialogues Clin Neurosci* 2003;5:115-128.

13. Schoepf D, Heun R. Anxiety disorders and physical comorbidity: increased prevalence but reduced relevance of specific risk factors for hospital-based mortality during a 12.5-year observation period in general hospital admissions. *Eur Arch Psychiatry Clin Neurosci* 2015;265:387-98 doi: 10.1007/s00406-014-0566-9 [published Online First 4 December 2014].

14. Player MS, Peterson LE. Anxiety disorders, hypertension, and cardiovascular risk: a review. *Int J Psychiatry Med* 2011;41:365-377 doi: https://doi.org/10.2190/PM.41.4.f [published Online First 26 October 2011].

15. de Waal MW, Arnold IA, Eekhof JA, van Hemert AM. Somatoform disorders in general practice: prevalence, functional impairment and comorbidity with anxiety and depressive disorders. *Br J Psychiatry* 2004;184:470-6 doi: 10.1192/bjp.184.6.470 [published Online First 1 June 2004].

16. Shen CC, Hu YW, Hu LY, et al. The risk of cancer in patients with generalized anxiety disorder: a nationwide population-based study. *PloS One* 2013;e57399 doi: 10.1371/journal.pone.0057399 [published Online First 27 February 2013].

17. Janszky I, Ahnve S, Lundberg I, Hemmingsson T. Early-onset depression, anxiety, and risk of subsequent coronary heart disease: 37-year follow-up of 49,321 young Swedish men. *J Am Coll Cardiol* 2010;56:31-37. doi: https://doi.org/10.1016/j.jacc.2010.03.033 [published Online First 22 June 2010].

18. Gale CR, Batty GD, Osborn DP, et al. Mental disorders across the adult life course and future coronary heart disease: evidence for general susceptibility. *Circulation* 2014;129:186-93 doi: 10.1161/CIRCULATIONAHA [published Online First 4 November 2013].

19. Carter RM, Wittchen HU, Pfister H, Kessler RC. One-year prevalence of subthreshold and threshold DSM-IV generalized anxiety disorder in a nationally representative sample. *Depress Anxiety* 2001;13:78-88.

20. Lamers F, van Oppen P, Comijs HC, et al. Comorbidity patterns of anxiety and depressive disorders in a large cohort study: the Netherlands Study of Depression and Anxiety (NESDA). *J Clin Psychiatry* 2011;72:341-8 doi: 10.4088/JCP.10m06176blu [published Online First 25 January 2011].

For peer review only

21. Johansson R, Carlbring P, Heedman A, Paxling B, Andersson G. Depression, anxiety and their comorbidity in the Swedish general population: point prevalence and the effect on health-related quality of life. *Peer J* 2013;1:e98 doi: 10.7717/peerj.98 [published Online First 9 July 2013].
22. Hamalainen J, Isometsa E, Sihvo S, Pirkola S, Kiviruusu O. Use of health services for major depressive and anxiety disorders in Finland. *Depress Anxiety* 2008;25:27-37 doi: 10.1002/da.20256 [published Online First 19 January 2007].
23. Maj M. 'Psychiatric comorbidity': an artefact of current diagnostic systems. *Br J Psychiatry* 2005;186:182-84 doi: 10.1192/bjp.186.3.182 [published Online First 28 February 2005].
24. Stein MB. Public health perspectives on generalized anxiety disorder. *J Clin Psychiatry* 2004;65:3-7.
25. Nutt D, Argyropoulos S, Hood S, et al. Generalized anxiety disorder: a comorbid disease. *Eur Neuropsychopharmacol* 2006;16:S109-18 doi: 10.1016/j.euroneuro.2006.04.003 [published Online First 5 June 2006].
26. Porensky EK, Dew MA, Karp JF, et al. The burden of late-life generalized anxiety disorder: effects on disability, health-related quality of life, and healthcare utilization. *Am J Geriatr Psychiatry* 2009;17:473-82 [published Online First 30 July 2012].
27. Munk-Jorgensen P, Allgulander C, Dahl AA, et al. Prevalence of generalized anxiety disorder in general practice in Denmark, Finland, Norway, and Sweden. *Psychiatr Serv* 2006;57):1738-44 doi: 10.1176/ps.2006.57.12.1738 [published Online First 28 February 2005].
28. Belanger L, Ladouceur R, Morin CM. Generalized anxiety disorder and health care use. *Can Fam Physician* 2005;51:1362-3.
29. Wittchen HU, Kessler RC, Beesdo K, et al. Generalized anxiety and depression in primary care: prevalence, recognition, and management. *J Clin Psychiatry* 2002;63:24.
30. Day N, Oakes S, Luben R, et al. EPIC-Norfolk: study design and characteristics of the cohort. European Prospective Investigation of Cancer. *Br J Cancer* 1999;80:95-103.
31. Hayat SA, Luben R, Keevil VL, et al. Cohort profile: a prospective cohort study of objective physical and cognitive capability and visual health in an ageing population

For peer review only

of men and women in Norfolk (EPIC-Norfolk 3). *Int J Epidemiol* 2014;43:1063-72 doi: 10.1093/ije/dyt086 [published Online First 14 June 2013].

32. Surtees P, Wainwright N, Khaw K, et al. Functional health status, chronic medical conditions and disorders of mood. *Br J Psychiatry* 2003;183**:**299–303 doi: 10.1192/bjp.183.4.299 [published Online First 30 September 2003].
33. Leng Y, Wainwright NWJ, Cappuccio FP, et al. Daytime napping and the risk of all-cause and cause-specific mortality: a 13-year follow-up of a British population. *Am J Epidemiol* 2014;179:1115-1124 doi: https://doi.org/10.1093/aje/kwu036 [published Online First 30 March 2014].
34. Leng Y, Wainwright NWJ, Cappuccio FP, et al. Self-reported sleep patterns in a British population cohort. *Sleep Med* 2014;15:295-302 doi: 10.1016/j.sleep.2013.10.015.
35. Leng Y, Wainwright NW, Cappuccio FP, et al. Daytime napping and increased risk of incident respiratory diseases: symptom, marker, or risk factor? *Sleep Med* 2016;23:12-15 doi: 10.1016/j.sleep.2016.06.012 [published Online First 12 July 2016].
36. Shohaimi S, Luben R, Wareham N, et al. Residential area deprivation predicts smoking habit independently of individual educational level and occupational social class. A cross sectional study in the Norfolk cohort of the European Prospective Investigation into Cancer (EPIC-Norfolk). *J Epidemiol Community Health* 2003;57:270-276 doi: 10.1136/jech.57.4.270.
37. Myint PK, Surtees PG, Wainwright NW, et al. Physical health-related quality of life predicts stroke in the EPIC-Norfolk. *Neurology* 2007;69:2243-8 doi: 10.1212/01.wnl.0000296010.21252.78.
38. Elias P, Halstead K, Prandy K. CASOC: Computer-assisted standard occupational coding. London, England: HMSO 1993.
39. Surtees PG, Wainwright NWJ, Brayne C. Psychosocial aetiology of chronic disease: a pragmatic approach to the assessment of lifetime affective morbidity in an EPIC component study. *J Epidemiol Community Health* 2000;54:114-122 doi: 10.1136/jech.54.2.114.
40. Remes O, Brayne C, Van der Linde R, et al. A systematic review of reviews on the prevalence of anxiety disorders in adult populations. *Brain Behav* 2016;6:e00497 doi: 10.1002/brb3.497 [published Online First 5 June 2016].

41. Ware JE, Snow K, Kosinski M, et al. New England Medical Center Hospital. Health Institute. SF-36 Health Survey: Manual and Interpretation Guide. The Health Institute; Boston, MA: New England Medical Center 1993.

42. Lakshman R, McConville A, How S, et al. Association between area level socio-economic deprivation and a cluster of behavioural risk factors: Cross sectional, population based study. *J Public Health (Oxf)* 2011;33:234-245. doi: 10.1093/pubmed/fdq072 [published Online First 29 September 2010].

43. Vetter S, Endrass J, Schweizer I, et al. The effects of economic deprivation on psychological well-being among the working population of Switzerland. *BMC Public Health* 2006;6:223. doi: 10.1186/1471-2458-6-223 [published Online First 4 September 2006].

44. NHS Digital. Adult Psychiatric Morbidity survey. http://content.digital.nhs.uk/catalogue/PUB21748 (accessed 29 September 2016).

45. NHS. Hospital Episode Statistics. http://content.digital.nhs.uk/hes (accessed 10 September 2017)

46. Luppa M, Sikorski C, Motzek T, et al. Health service utilization and costs of depressive symptoms in late life – a systematic review. *Curr Pharm Des* 2012;18:5936-57.

47. Ford JD, Trestman RL, Steinberg K, et al. Prospective association of anxiety, depressive, and addictive disorders with high utilization of primary, specialty and emergency medical care. *Soc Sci Med* 2004;58:2145-8 doi: 10.1016/j.socscimed.2003.08.017 [published Online First 25 September 2003].

48. Deacon B, Lickel J, Abramowitz JS. Medical utilization across the anxiety disorders. *J Anxiety Disord* 2008;22:344-50 doi: 10.1016/j.janxdis.2007.03.004 [published Online First 12 March 2007].

49. Klassen BJ, Porcerelli JH, Markova T. The effects of PTSD symptoms on health care resource utilization in a low-income, urban primary care setting. *J Trauma Stress* 2013;26:636-9 doi: 10.1002/jts.21838 [published Online First 12 September 2013].

50. Lehavot K, Der-Martirosian C, Simpson TL, et al. The role of military social support in understanding the relationship between PTSD, physical health, and healthcare utilization in women veterans. *J Trauma Stress*. 2013;26:772–5 doi: 10.1002/jts.21859 [published Online First 6 November 2013].

For peer review only

Figure 1 – Flowchart of EPIC-Norfolk cohort

This is a flowchart showing the number of participants at each study stage: the number approached to participate in the EPIC-Norfolk study, the number enrolled at baseline, and with complete data on all covariates. The EPIC-Norfolk study consists of middle-aged and older British people.

For peer review only

Figure 1 - Flowchart of EPIC-Norfolk cohort

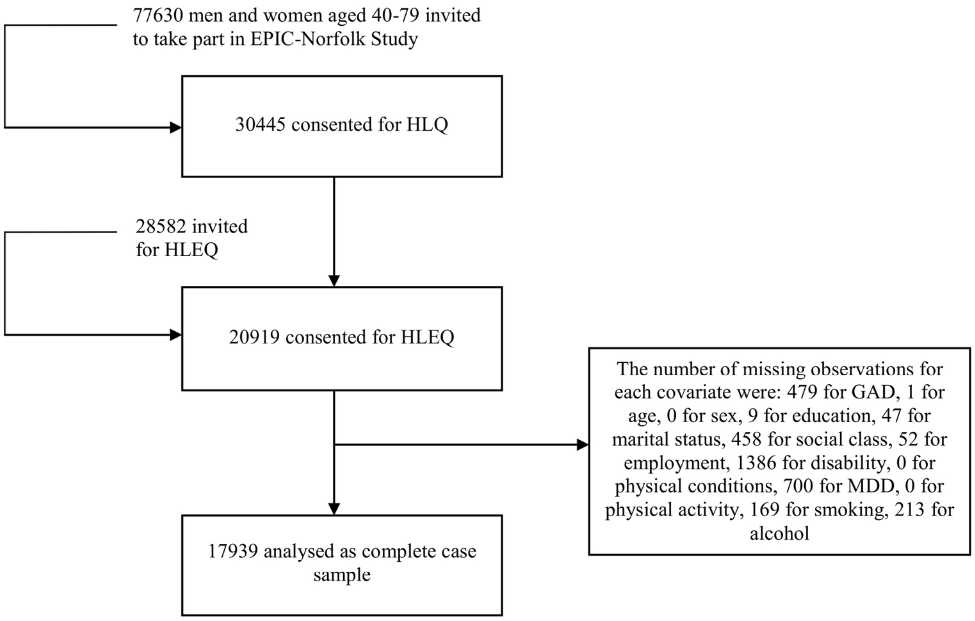

Note: Some participants had missing observations on more than one covariate.

Flowchart of the European Prospective Investigation of Cancer (EPIC)-Norfolk cohort. This is a flowchart showing the number of participants at each study stage: the number approached to participate in the EPIC-Norfolk study, the number enrolled at baseline, and with complete data on all covariates. The EPIC-Norfolk study consists of middle-aged and older British people.

153x135mm (300 x 300 DPI)

Appendix I: Table 1 Percentage and number of people with missing past-year GAD reported in 1996-2000 according to sociodemographic factors, health status, and behaviour risk factors for the EPIC-Norfolk cohort

Characteristic	Total number with characteristic	Percentage and no. with missing past-year GAD
**Socio-demographics**		
**Age (years)**		
<50	2385	1.1 (26)
50-60	6279	1.1 (70)
60-70	5787	0.9 (54)
70+	3685	1.3 (47)
**Sex**		
Women	10055	1.2 (118)
Men	8081	1.0 (79)
**Education**[‡]		
Low	6178	1.2 (72)
High	11958	1.1 (125)
**Marital status**		
Single	695	1.3 (9)
Married	14687	1.0 (149)
Other[*]	2754	1.4 (39)
**Social class**		
Manual	6918	1.2 (82)
Non-manual	11218	1.0 (115)
**Employment**		
Yes	7775	0.8 (63)[b]
No	10361	1.3 (134)
**Health status**		
**Physical conditions**[+]		
Yes	9285	1.3 (119)[b]
No	8851	0.9 (78)
**Disability level**		
High[¶]	9030	1.4 (130)[a]
Low	9106	0.7 (67)
**Psychiatric conditions**		
**Past-year MDD**		
Yes	983	5.0 (49)[a]
No	17153	0.9 (148)
**Behaviour risk factors**		
**Physical activity**		
Active[¥]	12963	1.1 (141)
Inactive	5173	1.1 (56)
**Smoking status**		
Current smoker	1922	1.5 (29)
Former smoker	7543	1.0 (73)

For peer review only

Never smoker	8671	1.1 (95)
**Alcohol intake**		
High[α]	9327	0.9 (86)[b]
Low	8809	1.3 (111)

‡ High education: O-level, A-level, degree; low education: refers to no education
* Other: divorced, separated, widowed
+ Physical conditions: respiratory disease (asthma and bronchitis), allergies and hay fever, stroke, heart attack, cancer, diabetes, thyroid conditions, arthritis
¶ Below the median PCS value of 50.6
¥ Moderately inactive, moderately active, active
α 3+ units of alc./week (1 pint beer=2 units, 1 glass wine=1 unit, 1 glass sherry=1 unit, 1 glass spirits=1 unit)

[a] $P < 0.001$
[b] $P < 0.05$

For peer review only

Appendix 2 – Multiple imputations for missing data

1. Main analysis: association between past-year GAD and non-psychiatric hospital admissions

First, we imputed missing data for all covariates except GAD, our main exposure. Based on the literature, we identified potential auxiliary variables, and we retained those that were correlated with the variables in our model and were good predictors of the missing status (based on statistical tests). Our imputation model included all variables from the analysis model and the auxiliary variables.

To retain as much information as possible, we conducted the imputations on non-transformed data-the original variables in our dataset. We imputed data using the fully conditional specification, and specified a linear regression model for continuous data that were normally distributed; predictive mean matching for continuous data that were not normally distributed; and logistic regression for categorical variables. Variable estimates were subsequently averaged from 100 imputed datasets using Rubin's rules (we transformed the data before running the analytic model of interest within each of the imputed datasets).[1]

We checked whether the imputations were acceptable by comparing 1) the means, standard deviations, and plots of recorded and imputed values for continuous variables, and 2) the frequencies and percentages of recorded and imputed values for each level of categorical variables.

Analyses were done using SAS 9·3 and p-values less than 0·05 were considered statistically significant.

Findings from this set of multiple imputations for missing data analysis provided the following effect estimate relating past-year GAD and non-psychiatric hospital admissions: IRR: 1.04, 95% CI: 1.02, 1.06. The IRR of 1.04 was the same as in the complete case analysis.

The same process was repeated and we imputed missing data for all covariates and also for GAD. This was the effect estimate: IRR: 1.05, 95% CI: 1.03, 1.08.

2. Subsidiary analysis: association between GAD-MDD comorbidity and non-psychiatric hospital admissions

When different forms of GAD were considered in relation to health service use, the effect estimate for GAD-MDD comorbidity was the only one that emerged as statistically significant in the complete case analysis. Therefore, we repeated this analysis using multiple imputations for missing data (similar process to that described in the first instance – we imputed missing data for all covariates except for GAD and MDD) and obtained the following result: IRR=1.20, 95% CI: 1.18, 1.22. The IRR is very similar to that obtained in the complete case analysis.

For peer review only

Auxiliary variables used in the imputation models

Variable	Questionnaire	Description of variable
**Sociodemographic factors**		
History of psychiatric illness	Health and Lifestyle Questionnaire (HLQ)	Self-reported history of other psychiatric illness
History of back pain	Health and Lifestyle Questionnaire (HLQ)	Self-reported history of back pain
History of cholesterol	Health and Lifestyle Questionnaire (HLQ)	Self-reported history of cholesterol
History of migraine	Health and Lifestyle Questionnaire (HLQ)	Self-reported history of migraine
History of tumour	Health and Lifestyle Questionnaire (HLQ)	Self-reported history of tumour

The questionnaire used for these variables have been previously described in the methods.

References

1. Berglund P, Heeringa S. Multiple imputation of missing data using SAS. Cary, NC: *SAS Institute Inc*, 2014.

For peer review only

Please see the article line numbers (column on the right) and the explanations provided.

STROBE Statement—checklist of items that should be included in reports of observational studies

	Item No	Recommendation	Line numbers within the article
**Title and abstract**	1	(*a*) Indicate the study's design with a commonly used term in the title or the abstract	9, 44
		(*b*) Provide in the abstract an informative and balanced summary of what was done and what was found	35-68
**Introduction**			
Background/rationale	2	Explain the scientific background and rationale for the investigation being reported	94-141
Objectives	3	State specific objectives, including any prespecified hypotheses	143-147
**Methods**			
Study design	4	Present key elements of study design early in the paper	155
Setting	5	Describe the setting, locations, and relevant dates, including periods of recruitment, exposure, follow-up, and data collection	154-165, 227-228
Participants	6	(*a*) *Cohort study*—Give the eligibility criteria, and the sources and methods of selection of participants. Describe methods of follow-up<br>*Case-control study*—Give the eligibility criteria, and the sources and methods of case ascertainment and control selection. Give the rationale for the choice of cases and controls<br>*Cross-sectional study*—Give the eligibility criteria, and the sources and methods of selection of participants	154-157, 159-161, 163-165, and especially 167-169
		(*b*) *Cohort study*—For matched studies, give matching criteria and number of exposed and unexposed<br>*Case-control study*—For matched studies, give matching criteria and the number of controls per case	
Variables	7	Clearly define all outcomes, exposures, predictors, potential confounders, and effect modifiers. Give diagnostic criteria, if applicable	Outcome: 222-245<br>Exposure: 171-181<br>Confounders: 182-220
Data sources/	8*	For each variable of interest, give sources of data and details of methods of	There were two variables of interest in this study:

For peer review only - http://bmjopen.bmj.com/site/about/guidelines.xhtml

measurement		assessment (measurement). Describe comparability of assessment methods if there is more than one group	health service use and generalized anxiety disorder (222-248, 171-181). The others are potential confounders – in the methods I list them all, indicate how they were assessed and mention that they were collected through the baseline, self-reported postal HLQ questionnaire.
Bias	9	Describe any efforts to address potential sources of bias	I conducted a complete case analysis, followed by multiple imputations of missing data.
Study size	10	Explain how the study size was arrived at	280-282
Quantitative variables	11	Explain how quantitative variables were handled in the analyses. If applicable, describe which groupings were chosen and why	How the variables were handled in analyses: 252-278<br><br>I mention that these groupings were based on the literature and provide the relevant citations.<br><br>How the outcome variable was created: 224-245<br>How GAD was created: 173-181<br>Covariates: 184-220; I mention that the final categorization of the variables was also done in accordance with previous literature and I cite the relevant studies.
Statistical methods	12	(*a*) Describe all statistical methods, including those used to control for confounding	250-278
		(*b*) Describe any methods used to examine subgroups and interactions	There were no subgroups or interactions examined. In addition to the main question (if past-year GAD leads to higher health service use), we also determined whether GAD frequency, chronicity, age at onset, and comorbidity with MDD are associated with health service use – this is explained in lines 271-278.
		(*c*) Explain how missing data were addressed	We did a complete-case analysis followed by multiple imputations for missing data.
		(*d*) *Cohort study*—If applicable, explain how loss to follow-up was addressed<br>*Case-control study*—If applicable, explain how matching of cases and controls was	Loss to follow-up was not a problem in this study. We were able to track down all participants using

For peer review only - http://bmjopen.bmj.com/site/about/guidelines.xhtml

addressed<br>*Cross-sectional study*—If applicable, describe analytical methods taking account of sampling strategy	various means, unless they expressed that they wished to be removed from the mailing list. We elaborate on this in the manuscript.
(*e*) Describe any sensitivity analyses	

For peer review only

For peer review only - http://bmjopen.bmj.com/site/about/guidelines.xhtml

**Results**			
Participants	13*	(a) Report numbers of individuals at each stage of study—eg numbers potentially eligible, examined for eligibility, confirmed eligible, included in the study, completing follow-up, and analysed	
		(b) Give reasons for non-participation at each stage	We do not have the reasons for non-participation, because these data were not collected when the study was initiated in 1993.
		(c) Consider use of a flow diagram	Flow diagram included in submission – figure 1.
Descriptive data	14*	(a) Give characteristics of study participants (eg demographic, clinical, social) and information on exposures and potential confounders	301-302, 320-324, 341-344, as well as, tables 1 and 2
		(b) Indicate number of participants with missing data for each variable of interest	See flow diagram.
		(c) *Cohort study*—Summarise follow-up time (eg, average and total amount)	298-299
Outcome data	15*	*Cohort study*—Report numbers of outcome events or summary measures over time	Table 2 provides the mean number of hospital admissions for those with/without GAD over the 9-year follow-up.
		Case-control study—Report numbers in each exposure category, or summary measures of exposure	
		Cross-sectional study—Report numbers of outcome events or summary measures	
Main results	16	(*a*) Give unadjusted estimates and, if applicable, confounder-adjusted estimates and their precision (eg, 95% confidence interval). Make clear which confounders were adjusted for and why they were included	Tables 3 and 4 contain unadjusted and progressively adjusted estimates. We also discussed the findings within the text, and provide odds ratios and 95% confidence intervals.<br><br>We included the confounders based on the literature – we mention this in the paper and cite relevant literature. As per strobe, we included this information in the methods section; and we omitted repeating this in the results section to reduce redundancy. However, if the editor would like us to repeat this information in the results, we are happy to do so.

For peer review only - http://bmjopen.bmj.com/site/about/guidelines.xhtml

		(*b*) Report category boundaries when continuous variables were categorized	The cut-offs for age, alcohol intake, and disability levels are provided (see also footnotes of tables).
		(*c*) If relevant, consider translating estimates of relative risk into absolute risk for a meaningful time period	
Other analyses	17	Report other analyses done—eg analyses of subgroups and interactions, and sensitivity analyses	The Results section contains the findings from the multiple imputations for missing data analysis – we also included further information on this in an appendix. Findings for GAD frequency, chronicity, age of onset, and comorbidity with MDD are reported in table 4.
**Discussion**			
Key results	18	Summarise key results with reference to study objectives	415-423
Limitations	19	Discuss limitations of the study, taking into account sources of potential bias or imprecision. Discuss both direction and magnitude of any potential bias	439-477
Interpretation	20	Give a cautious overall interpretation of results considering objectives, limitations, multiplicity of analyses, results from similar studies, and other relevant evidence	514-546 (We also have a section comparing our study results to those of others: 479-499, as well as a section on potential mechanisms explaining our findings: 501-512)
Generalisability	21	Discuss the generalisability (external validity) of the study results	472-474, 460-469
**Other information**			
Funding	22	Give the source of funding and the role of the funders for the present study and, if applicable, for the original study on which the present article is based	562-563, 579-581

*Give information separately for cases and controls in case-control studies and, if applicable, for exposed and unexposed groups in cohort and cross-sectional studies.

Note: An Explanation and Elaboration article discusses each checklist item and gives methodological background and published examples of transparent reporting. The STROBE checklist is best used in conjunction with this article (freely available on the Web sites of PLoS Medicine at http://www.plosmedicine.org/, Annals of Internal Medicine at http://www.annals.org/, and Epidemiology at http://www.epidem.com/). Information on the STROBE Initiative is available at www.strobe-statement.org.

For peer review only

For peer review only - http://bmjopen.bmj.com/site/about/guidelines.xhtml
